# Sox17 is required for endothelial regeneration following inflammation-induced vascular injury

**DOI:** 10.1038/s41467-019-10134-y

**Published:** 2019-05-09

**Authors:** Menglin Liu, Lianghui Zhang, Glenn Marsboom, Ankit Jambusaria, Shiqin Xiong, Peter T. Toth, Elizaveta V. Benevolenskaya, Jalees Rehman, Asrar B. Malik

**Affiliations:** 1 Department of Pharmacology, The University of Illinois College of Medicine, Chicago, IL 60612 USA; 2 Department of Biochemistry and Molecular Genetics, The University of Illinois College of Medicine, Chicago, IL 60607 USA; 3 Department of Medicine, Division of Cardiology, The University of Illinois College of Medicine, Chicago, IL 60612 USA; 4 University of Illinois Cancer Center, The University of Illinois at Chicago, Chicago, IL 60612 USA

**Keywords:** Sepsis, Vascular diseases, Regeneration

## Abstract

Repair of the endothelial cell barrier after inflammatory injury is essential for tissue fluid homeostasis and normalizing leukocyte transmigration. However, the mechanisms of endothelial regeneration remain poorly understood. Here we show that the endothelial and hematopoietic developmental transcription factor Sox17 promotes endothelial regeneration in the endotoxemia model of endothelial injury. Genetic lineage tracing studies demonstrate that the native endothelium itself serves as the primary source of endothelial cells repopulating the vessel wall following injury. We identify Sox17 as a key regulator of endothelial cell regeneration using endothelial-specific deletion and overexpression of Sox17. Endotoxemia upregulates Hypoxia inducible factor 1α, which in turn transcriptionally activates Sox17 expression. We observe that Sox17 increases endothelial cell proliferation via upregulation of Cyclin E1. Furthermore, endothelial-specific upregulation of Sox17 in vivo enhances lung endothelial regeneration. We conclude that endotoxemia adaptively activates Sox17 expression to mediate Cyclin E1-dependent endothelial cell regeneration and restore vascular homeostasis.

## Abstract (web summary)

Endothelial cell regeneration is essential for blood vessels to recover from inflammation-induced injury. Here Liu et al. show that the transcription factor Sox17 is required for endothelial regeneration following endotoxemia, and that delivery of a transgene expressing Sox17 to lung endothelial cells enhances recovery after injury.

## Introduction

Transport of solutes, plasma proteins and liquid and trafficking immune cells across the intact endothelial barrier is essential for tissue homeostasis^1,2^. Injury or death of endothelial cells (ECs) results in breakdown of the EC barrier, tissue edema, and the unfettered influx of inflammatory cells^1,3^. Endothelial regeneration is therefore an essential adaptive response^4,5^. In inflammatory diseases such as acute lung injury (ALI), which is characterized by  severe endothelial injury^6,7^, the inflammatory injury often exceeds the regenerative capacity of the endothelium. Thus, regeneration of the endothelium using stem or progenitor cells has emerged as a potential strategy for restoring tissue homeostasis^8–10^. However, the prospect of EC regeneration through transplantation of exogenous regenerative cells remains challenging due to the limited engraftment^11,12^ and intimal retention^13^ of the regenerative cells. An alternative approach is to activate intrinsic EC regeneration mechanisms. But little is known about the signaling mechanisms or cells responsible for endothelial regeneration following severe inflammatory injury^14–16^.

Activation of developmental pathways may be key to activating endothelial regeneration, as shown in the liver and thymus where re-activation of the developmental transcription factors Id1, Foxn1, and Notch promote regeneration^17–19^. Sox17, a member of the Sry-related high mobility group domain family F (SoxF) transcription factors^20^, is a key developmental regulator of endothelial and hematopoietic lineages^21–23^. Sox17 expression in mouse embryonic and adult arterial ECs is also an important mechanism of arterial integrity^24,25^. In addition, Sox17 induces angiogenesis in tumors^26^ and participates in the conversion of fibroblasts to reparative ECs^27^.

In the present study, using a mouse endotoxemia model of inflammatory endothelial injury, we demonstrate that endothelial Sox17 expression plays an obligatory role in normalizing the endothelium. Restoration of endothelial integrity is mediated by activation of Hypoxia inducible factor-1α (HIF-1α), upregulation of its target Sox17, and subsequent downstream expression of Cyclin E1 which mediates endothelial regeneration.

## Results

### Lineage tracing analysis of EC regeneration in vivo

**Fig. 1:**
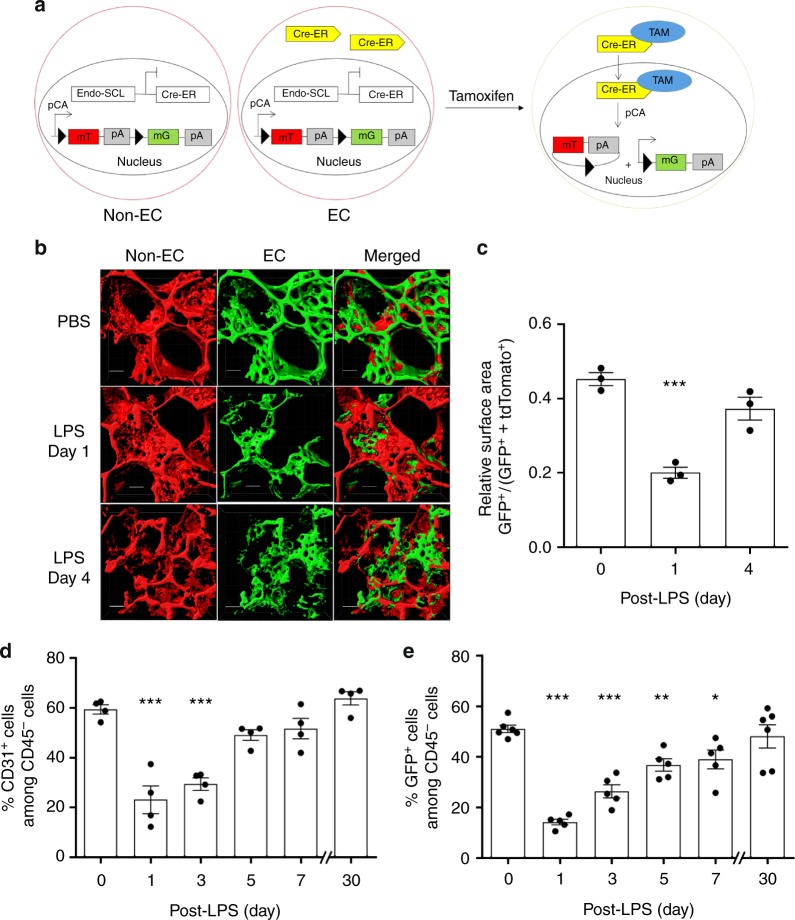
Lineage tracing analysis of lung EC injury induced by endotoxemia and kinetics of regeneration. **a** We carried out studies to establish a model of endotoxemia (LPS) induced EC injury in lungs followed by progressive recovery of endothelium. Studies were made in mTmG double fluorescent lineage tracing mice using endothelial-enhanced *Scl*-CreERT2. After tamoxifen induction, Cre recombinase in ECs translocates to the nucleus and induces EGFP expression. **b** 2 photon imaging and 3D surface reconstruction of lungs from mTmG-*Scl* mice at baseline and days post-LPS-induced vascular injury (LPS is given i.p. at a dose of sub-lethal 12 mg/kg i.p.). Red indicates non-ECs and green indicates ECs. Scale bar = 20 μm. *n* = 3. Same lung sample in 3D structure movie were shown in Videos S1 and S2 for baseline and post-injury day 1. **c** Quantification of the surface area of EGFP^+^ (EGFP positive) cells relative to total surface areas (EGFP^+^ TdTomato). EGFP^+^ cell surface area, reflecting EC population, is 50% of total lung cell population at baseline. The EC population significantly decreases at post-LPS day 1 and progressively recovers by day 4. *n* = 3. **d** Flow cytometry analysis of ECs shown as percent of CD31^+^CD45^−^ (CD31 positive, CD45 negative) cells. *n* = 4. In the non-leukocyte fraction (exclusion of CD45^+^ cells), CD31^+^ ECs, were significantly reduced within first the 24 h post-LPS-induced injury and then gradually recovered within 7 days. **e** Flow cytometry analysis of EGFP^+^ cells percentage among whole lung population. *n* = 5. Similar to **d**, EGFP^+^ cells also showed a marked loss of ECs induced by LPS within the first 24 h and then a full restored population by day 7 post-LPS. *P < 0.05 and ***P < 0.001 versus day 0. Data are shown as mean ± SEM. Analysis was performed using one-way ANOVA

To address mechanisms of EC regeneration, we first carried out EC lineage tracing analysis to assess the kinetics of the EC injury and repair response. For these studies, we crossbred dual-fluorescent color mTmG reporter mice^28^ with EC-specific *Scl*-CreERT2 mice to generate an EC specific genetic lineage tracing mouse model. In these *Scl*-CreERT2 mice, the tamoxifen-inducible Cre recombinase is driven by a 5′ endothelial enhancer of the stem cell leukemia (*Scl*) locus that is restricted to the endothelium. This is in contrast to the wild-type *Scl* locus that is also expressed in hematopoietic cells^29^. The mTmG double-fluorescent reporter mice express membrane-targeted tdTOMATO (mT) prior to Cre-mediated excision and membrane-targeted enhanced GFP (mG) after excision in ECs (Fig. 1a). This transgenic mouse model showed ~80% labeling efficiency and ~95% EC specificity (Supplementary Fig. 1A). Using 2-photon imaging of lungs in live mice, we observed that a sublethal concentration of the bacterial endotoxin lipopolysaccharide (LPS) i.p. (12 mg/kg), which induces severe inflammatory injury, produced acute and severe loss of EGFP^+^ (EGFP positive) ECs; this was followed by a period of gradual recovery over several days (Fig. 1b, Supplementary Videos 1 and 2). Quantification showed decreased surface area of EGFP^+^ ECs at day 1 post-LPS and then recovery over 4 days (Fig. 1c). We also quantified the percentage of ECs derived from resident ECs (EGFP^+^ ECs) using flow cytometry. Freshly isolated lung ECs from mice were immunostained with the EC marker CD31 and the leukocyte marker CD45 was used to exclude CD45^+^ leukocytes, because they can also co-express CD31 (Supplementary Fig. 1B). The EC population (defined as CD31 positive, CD45 negative (CD31^+^CD45^−^) cells) decreased markedly as a percentage of total cell lung population at day 1 post-LPS but then fully recovered by day 7 (Fig. 1d). EGFP^+^ ECs, reflecting the native endothelium, showed the same decrease in the EC population and subsequent regeneration (Fig. 1e). By day 7, the EGFP^+^ cell percentage reached the pre-injury levels and remained stable thereafter. These results defined both the time course of loss of ECs in the endotoxemia model of EC injury and recovery as well as the central role of the native endothelium in endothelial regeneration.

### Endotoxin upregulates Sox17 expression in vivo

**Fig. 2:**
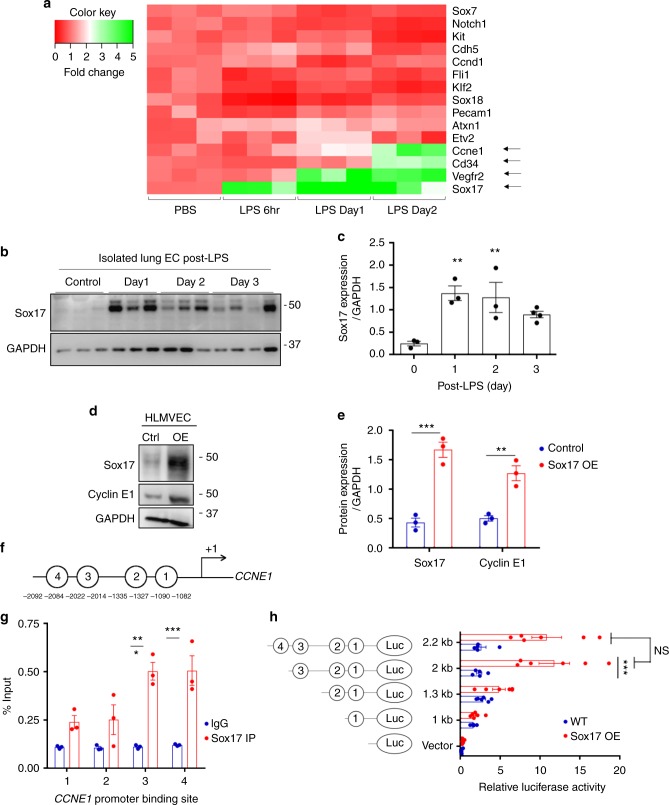
Activation of Sox17 at onset of EC regeneration and Sox17-mediated Cyclin E1 expression. **a** qPCR analysis of gene expression in sorted CD31^+^ cells from mTmG-*Scl* mice before and after injury (12 mg/kg i.p.). *Sox17*, *Vegfr2*, and *Ccne1* increased significantly at day 2 post-LPS compared to baseline. *n* = 3. Color scale: the fold change increases from red to white to green color. **b** Western blot analysis in fresh isolated ECs from wild-type mice and quantification **c** showed a 5-fold increase in Sox17 protein expression within 1 day following injury compared to baseline and followed by recovery within 3 days post-LPS. *n* = 3. **d**, **e** Western blot analysis of cultured HLMVECs in which Sox17 was overexpressed showed 2.5x fold increase in Cyclin E1 protein expression relative to control cells. *n* = 3. OE, overexpression. **f** Representation of the *CCNE1* promoter region with Sox17 binding sites (circled numbers) and their sequences. **g** HLMVECs were retrovirally transduced with Sox17 or control plasmid for 3 days, and Ch-IP assay followed by qPCR was performed to amplify Sox17 binding sites in the *CCNE1* promoter. *n* = 3. **h** 293T cells were transfected with a Sox17 overexpression plasmid containing *CCNE1* luciferase reporter constructs. Luciferase values were normalized to Renilla luciferase control reporter values. A schematic representation of corresponding deletion constructs is presented in the right panel. *n* = 3 and duplicates per sample. **P < 0.01 and ***P < 0.001. Data are shown as mean ± SEM. Analysis was performed using one-way ANOVA for (**c**) and two-way ANOVA with Bonferroni post-tests for (**e**, **g**, **h**)

To investigate the mechanisms of EC regeneration, we performed qPCR analysis of gene expression in sorted CD31^+^ ECs obtained from mTmG-*Scl* mice at 6 hrs, 1 day and 2 days post-LPS challenge (12 mg/kg LPS i.p.) with PBS-injected mice serving as controls. The heat map of mRNA expression depicting key genes involved in endothelial development and growth revealed that *Vegfr2*, *Sox17*, and *Ccne1* were significantly upregulated at post-LPS day 2 compared to baseline (Fig. 2a). Importantly, *Sox17* expression increased as early as 6 h following injury whereas *Vegfr2* and *Ccne1* expression was increased at later time points. Because Sox17 is known to regulate vascular development^30^, we focused on its possible role in EC regeneration. Immunoblotting of freshly isolated murine lung ECs showed that SOX17 protein expression increased 5-fold post-LPS in ECs as compared to controls (Fig. 2b, c). We also determined expression of the transcription factor ER71 linked to angiogenesis and regeneration^31^ as a potential mechanism of EC regeneration, and found its protein expression did not increase following LPS (Supplementary Fig. 2). To determine next whether Sox17 contributed to regeneration through EC proliferation, we determined the kinetics of Cyclin E1 gene and protein expression known to regulate cell cycle activity^32^. We observed that *Ccne1* was upregulated at Day 2 post-LPS (Fig. 2a). Furthermore, immunoblotting of human lung microvascular endothelial cells (HLMVECs) showed markedly increased Cyclin E1 protein expression in the cells over-expressing Sox17 as compared to control cells (Fig. 2d, e). We also identified several putative Sox17 binding sites in the *CCNE1* promoter using the Sox17 binding sequence^33^ from the JASPAR database (Fig. 2f and Supplementary Fig. 3). Chromatin immunoprecipitation (Ch-IP) was carried out in HLMVECs overexpressing with Sox17. qPCR of the 4 Sox17 identified binding sites showed that Sox17 bound only to sites 3 and 4 on *CCNE1* promoter (Fig. 2g). Using luciferase promoter activity reporter assay, we observed that Sox17 binding site 3 in the *CCNE1* promoter increased luciferase activity by 5-fold in contrast to other binding sites (Fig. 2h). Thus, Sox17 binding site 3 in the *CCNE1* promoter appears to be critical for Sox17-mediated transactivation of *CCNE1*. Together these results show that LPS-induced EC injury increases Sox17 expression and Sox17 induces EC proliferation via upregulating Cyclin E1.

### EC-specific Sox17 deletion prevents EC regeneration

**Fig. 3:**
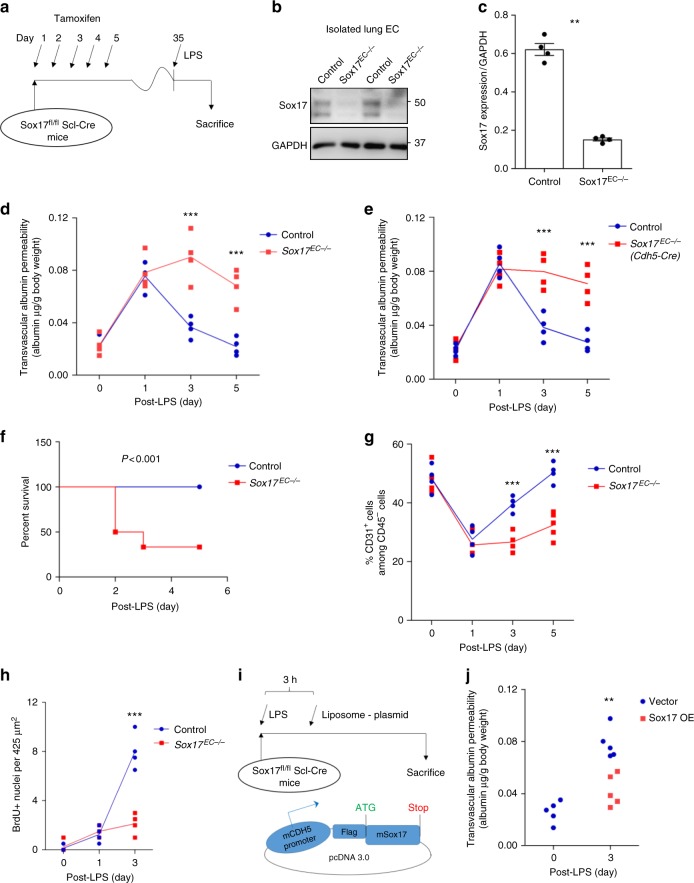
EC specific deletion of Sox17 (*Sox17*^EC−/−^) in mice prevents endothelial regeneration. **a** Schematic diagram of *Sox17*^*fl/fl*^ mice crossed with *Scl*-CreERT2 to delete Sox17 in ECs. After tamoxifen feeding for five days and rest for another four weeks, mice are challenged with LPS (sub-lethal 8 mg/kg, i.p.) for analysis. **b** Western blot analysis of Sox17 protein expression in isolated ECs obtained from flushed lungs of *Sox17*^EC−/−^ and control mice. *n* = 4. **c** Quantification of **b** shows 80% deletion of Sox17 in ECs of *Sox17*^EC−/−^ mice compared to control mice. *n* = 4. **d** Time course of lung transvascular permeability following LPS challenge in *Sox17*^EC−/−^ and *Sox17*^*fl/fl*^ mice. *n* = 4. While control mouse lungs showed increased endothelial permeability at day 1 post-LPS and then recovered to baseline by day 5, *Sox17*^EC−/−^ mice showed prolonged endothelial barrier leakiness post-LPS. **e** Time course of changes in lung transvascular permeability following LPS challenge was also carried out in *Sox17*^*fl/fl*^ mice crossed with *Cdh5*-CreERT2 mice. *n* = 4. Similar as in **d**, these *Sox17*^EC−/−^ mice also showed persistent leakiness post-LPS while control mice fully recovered. **f** Survival curve of LPS challenge in *Sox17*^EC−/−^ and control mice. *n* = 8 per group. At this sub-lethal dose, all control mice survived whereas half of *Sox17*^EC−/−^ mice died on day 2 post-LPS with increased mortality on day 3. By day 5, the death rate for control mice is 0 while for *Sox17*^EC−/−^ mice is 60%. **g** Flow cytometry analysis of CD31^+^CD45^−^ ECs among whole lung population in mice following injury. *n* = 4. In contrast to control mice in which CD31^+^CD45^−^ EC population gradually recovered with day 3 post-LPS after initial loss of ECs, *Sox17*^EC−/−^ mice showed significantly delayed restoration of ECs post-LPS period. **h** Quantification of BrdU+ nuclei in each field of 425 μm^2^ area in flushed lung cryo-sections from mice following injury. *n* = 4 mice per group and 6 replicates per sample. Slides are co-stained with CD31-AF488, BrdU-APC, and DAPI. At day 3 post-LPS, the control group showed a significantly higher number of BrdU+ECs compared to baseline. However, *Sox17*^EC−/−^ mice showed markedly reduced level of BrdU+ECs, indicating reduced EC proliferation. **i** To assess whether expression of Sox17 in ECs can restore lung endothelial integrity, studies were performed in *Sox17*^EC−/−^ mice to overexpress Sox17 protein. We used a mixture of 50 μg plasmid (mouse *Cdh5* promoter—Flag —Sox17) encapsulated in liposomes, which were injected i.v. 3 h after LPS challenge. **j** At day 3 post-LPS, liposome vector-treated *Sox17*^EC−/−^ mice showed marked EC barrier leakiness as assessed by lung transvascular permeability of albumin whereas the Sox17-rescued mice showed markedly reduced endothelial permeability. *n* = 4. OE, overexpression. *P < 0.05, **P < 0.01 and ***P < 0.001. Data are shown as mean ± SEM. Analysis was performed using two-tailed Student’s *t*-test for (**c**, **j**), two-way ANOVA with Bonferroni post-tests for (**d**, **e**, **g**, **h**) and Log-rank (Mantel-Cox) test for (**f**)

We next deleted Sox17 in ECs by crossing *Sox17*^*fl/fl*^ mice (Jackson Laboratory) with the tamoxifen-inducible endothelial-specific endo-*Scl*-Cre-ERT2 mice^29^. Tamoxifen (80mg/kg) was given daily for five consecutive days and experiments were carried out after four weeks when EC-expressed SOX17 was deleted (Fig. 3a). Immunoblotting confirmed the deletion in *Sox17*^*EC−/−*^ mice compared to *Sox17*^*fl/fl*^ mice (Fig. 3b, c). We observed that 12 mg/kg LPS induced 40–50% lethality in wild-type mice. Since Sox17 regulates endothelial proliferation, we surmised that the LPS dosage of 12 mg/kg in *Sox17*^*EC−/−*^ mice would induce even greater mortality, and therefore lowered LPS to sublethal dose of 8 mg/kg in order to compare responses in *Sox17*^*fl/fl*^ vs. *Sox17*^*EC−/−*^ mice. We found that LPS increased lung vascular permeability at day 1 post-LPS with no significant difference between *Sox17*^*EC−/−*^ and *Sox17*^*fl/fl*^ mice (Fig. 3d); thus, Sox17 did not affect severity of the initial LPS-induced endothelial injury. However, measurements of lung vascular leak during the recovery phase showed the critical role for endothelial Sox17 in mediating EC regeneration following the inflammatory injury. *Sox17*^*EC−/−*^ mice exhibited persistently increased endothelial permeability (Fig. 3d), indicative of delayed EC regeneration response. To confirm this finding, we also generated a second EC-specific Sox17 deletion mouse model using Cre driven by *Cdh5* promoter. These mice were generated by breeding *Cdh5*-CreERT2 mice^34^ with *Sox17*^*fl/fl*^ mice. We observed in these mice the same changes seen in endo-*Scl*-CreERT2 mice (Fig. 3e). All *Sox17*^*fl/fl*^ mice survived the sublethal LPS dosage (8 mg/kg i.p.) while 60% of the *Sox17*^*EC−/−*^ mice died (Fig. 3f). FACS analysis of freshly isolated lung ECs showed that *Sox17*^*EC−/−*^ and *Sox17*^*fl/fl*^ mice both had severe loss of EC at day 1 post-LPS. By day 5, however, the EC population was restored to baseline in *Sox17*^*fl/fl*^ mice but EC regeneration was severely defective in *Sox17*^*EC−/−*^ mice (Fig. 3g). This finding paralleled the significantly higher mortality seen in EC-specific Sox17 deleted mice. Next to assess EC proliferation, the nucleotide analog 5-bromo-2′-deoxyuridine (BrdU) was injected (i.p.) 14 h before sacrifice. Immunohistochemistry and confocal microscopy showed significantly increased BrdU^+^ ECs in *Sox17*^*fl/fl*^ mice at day 3 post-LPS when compared to *Sox17*^*EC−/−*^ mice (Fig. 3h and Supplementary Fig. 4).

We next determined whether re-expression of Sox17 in ECs of *Sox17*^*EC−/−*^ mice is sufficient to restore lung endothelial integrity. These studies were conducted in *Sox17*^*EC−/−*^ mice in which the Sox17 protein was expressed in lung ECs via i.v. liposomal delivery^35–37^. The Flag–*Sox17* plasmid was driven by EC-specific mouse *Cdh5* promoter to maximize its expression in ECs; and the effectiveness of the approach was determined (Supplementary Fig. 7), demonstrating marked augmentation of Sox17 expression in ECs isolated from the lungs during the days following i.v. delivery of liposomal *Sox17* with an EC-specific promoter but no such augmentation of Sox17 expression was found in the non-EC fraction. Liposomes encapsulating Flag–*Sox17* cDNA were injected i.v. 3 h after LPS challenge in mice (Fig. 3i). We observed that increased Sox17 expression in *Sox17*^*EC−/−*^ mice significantly accelerated endothelial barrier restoration compared to control vector delivery (Fig. 3j).

**Fig. 4:**
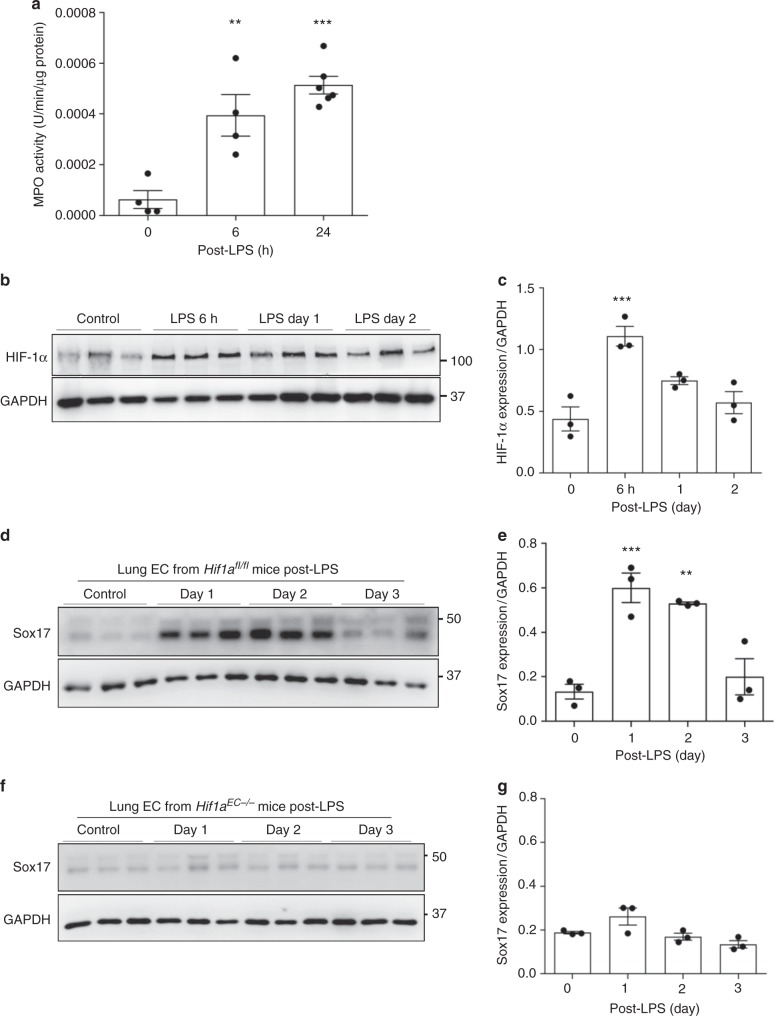
soHIF-1α signaling induces Sox17 expression. **a** MPO activity of flushed lung sample from mice challenged with LPS (12 mg/kg i.p.) for 6 and 24 h. *n* = 3. **b** Western blot analysis of wild-type mice lung before and after LPS-induced injury (12 mg/kg i.p.) and its quantification **c** showed that HIF-1α protein expression increased within 6 h post-LPS and remained increased until day 2. *n* = 3. **d** Western blot analysis in freshly isolated ECs from *Hif1a*^*fl/fl*^ mice and quantification **e** showed significantly increased Sox17 protein expression after injury when compared to baseline levels. *n* = 3. **f** Western blot analysis in freshly isolated ECs from *Hif1a*^EC−/−^ mice and quantification **g** showed no significant difference in Sox17 protein expression before and after injury. *n* = 3. **P < 0.01 and ***P < 0.001. Data are shown as mean ± SEM. Analysis was performed using one-way ANOVA for (**b**, **c**, **e**, **g**)

We next addressed mechanisms of upstream regulation of Sox17 expression in ECs. A key feature of endotoxin-induced inflammatory lung injury is widespread influx of activated neutrophils which intensely consume oxygen and the induce relative tissue hypoxia^38,39^. We thus assessed neutrophil infiltration in lungs by measuring myeloperoxidase (MPO) activity and observed 6-fold increase in MPO activity within 6 h post-LPS challenge and which continued to increase up 24 h (Fig. 4a). We assessed whether the hypoxia-responsive transcription factor HIF-1α^40,41^ was concomitantly increased, and observed its protein upregulation in the timeframe of neutrophil infiltration (Fig. 4b, c).

To study the relationship between HIF-1α and Sox17 in vivo, we deleted HIF-1α in an EC-specific manner by crossing *Hif1a*^*fl/fl*^ mice with Tie2-CreERT2 mice. At baseline EC-specific HIF-1α deletion in mice did not affect SOX17 expression (Supplementary Fig. 8). Immunoblotting showed that while SOX17 expression significantly increased in ECs post-LPS in *Hif1a*^*fl/fl*^ mice (Fig. 4d, e), SOX17 expression did not change either before or after LPS in *Hif1a*^*EC−/−*^ mice (Fig. 4f, g); thus, EC specific deletion of HIF-1α prevented the increase in endothelial Sox17 expression following LPS induced EC injury.

### HIF-1α signaling in ECs activates Sox17 transcription and expression

**Fig. 5:**
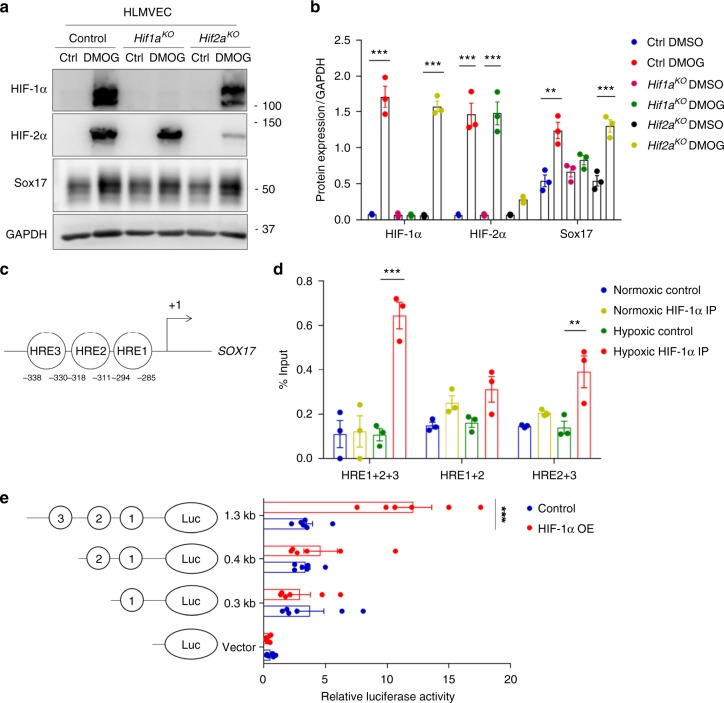
HIF-1α activates transcription of Sox17. **a** Western blot analysis in control HLMVECs and HLMVECs for which CRISPR/Cas9 was used to delete HIFs. ECs were treated with the HIF prolyl hydroxylase inhibitor DMOG to induce HIF expression. DMOG (1 mM) increased HIF-1α and HIF-2α protein expression in control ECs but not in ECs lacking HIF-1α or HIF-2α. Induction of HIF expression was coupled to Sox17 upregulation. *n* = 3. **b** Quantification of **a** showed that protein expression of HIF-1α and HIF-2α in ECs was significantly increased by DMOG treatment. Sox17 showed a 2.5-fold increase in Sox17 expression control DMSO treated ECs. The increase in Sox17 was significantly reduced in HIF-1α-deleted ECs but preserved in HIF-2α-deleted ECs, indicating the importance of HIF-1α in mediating Sox17 expression. *n* = 3. **c** Representation of the *SOX17* promoter region with 3 HREs indicated by circled numbers, and their respective sequences are displayed. **d** HLMVECs were exposed to normoxia or 1% O2 (hypoxia) for 8 h. Ch-IP assay followed by qPCR was performed to amplify the HRE regions in the *SOX17* promoter. Studies were performed in ECs exposed to either normoxia or hypoxia. *n* = 3. **e** 293T cells were transfected with a HIF-1α overexpression plasmid containing *SOX17* luciferase reporter constructs. Luciferase values were normalized to Renilla luciferase control reporter values. A schematic representation of corresponding deletion constructs is presented in the right panel. *n* = 3 and duplicates per sample. Results show that hypoxia activation of *SOX17* HRE3 was required for Sox17 expression. **P < 0.01 and ***P < 0.001. Data are shown as mean ± SEM. Analysis was performed using two-way ANOVA with Bonferroni post-tests for (**b**, **d**, **e**)

To examine the role of HIF-activation in regulating Sox17 expression, we designed specific guide RNAs to delete either HIF-1α (*Hif1a*^*KO*^) or HIF-2α (*Hif2a*^*KO*^) in HLMVECs using CRISPR/Cas9^42^. Dimethyloxalylglycine (DMOG), a prolyl-4-hydroxylase inhibitor^43,44^, was added to HLMVECs to stabilize both HIFs. We observed that HIF-1α, HIF-2α, and SOX17 protein expression was significantly increased after DMOG treatment as compared to control (Fig. 5a, b). Importantly, CRISPR/Cas9 mediated deletion of HIF-1α prevented the increase of SOX17 induced by DMOG whereas HIF-2α deletion did not (Fig. 5a, b), indicating the essential role of HIF-1α in mediating Sox17 expression.

On analyzing the promoter region of *SOX17* for hypoxia response elements (HREs), we found 3 sites (Fig. 5c and Supplementary Fig. 5A), one of which is conserved across multiple species (HRE1, −294 ~ −285) (Supplementary Fig. 5B). Chromatin immunoprecipitation (Ch-IP) was carried out to determine binding of HIF-1α to *SOX17* promoter using HLMVECs collected after 8 h of hypoxia (1% O_2_) with 21% O_2_ as the normoxic control. qPCR of amplified HRE regions showed that HIF-1α bound to HREs in the *SOX17* promoter (Fig. 5d and Supplementary Fig. 5C). We observed using a luciferase promoter activity reporter assay that only HRE3 significantly increased luciferase activity  upon co-expression with a degradation-resistant HIF-1α construct (Fig. 5e).

### Sox17 expression regenerates ECs and enhances survival in mice

**Fig. 6:**
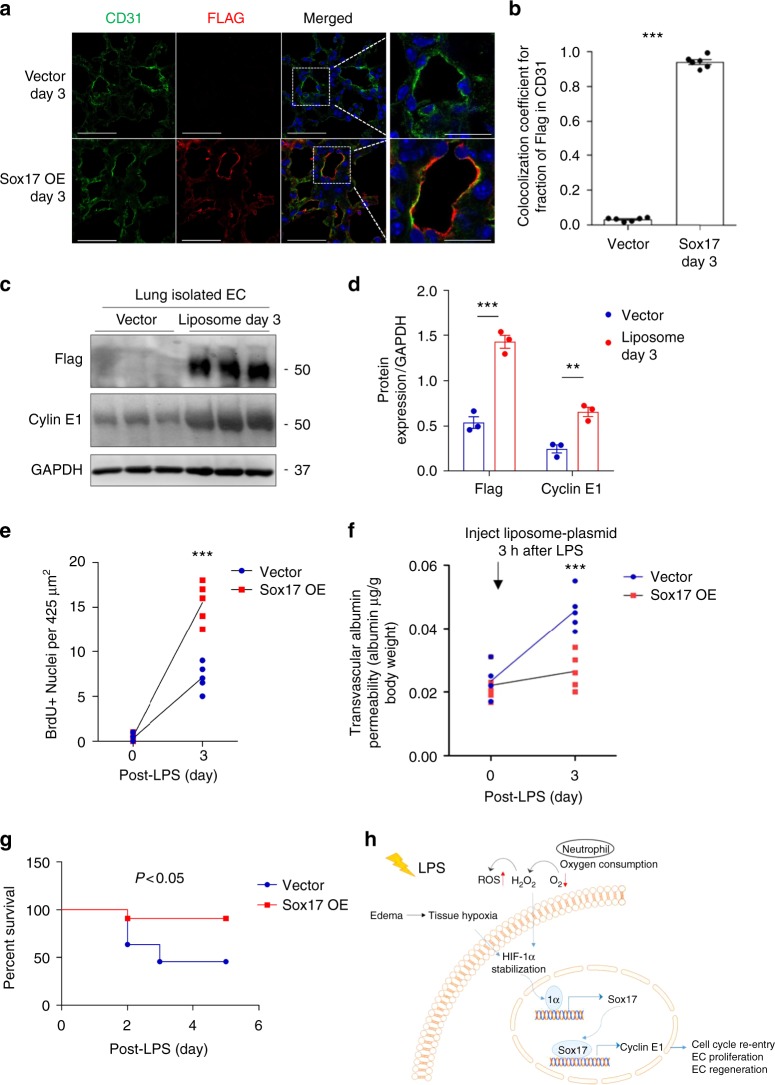
Overexpression of Sox17 in ECs induces EC proliferation and regeneration. Mixture of 50 μg plasmid with 100 μl liposomes was injected i.v. 3 h after LPS challenge (12 mg/dose i.p.) in wild-type mice. This plasmid has a Flag-tag added to the N-terminus of *Sox17* protein coding region and expression is under the regulation of a mouse *Cdh5* promoter. **a** Confocal microscopy of flag staining with CD31 and DAPI co-staining for nuclei in lung cryo-sections from mice receiving a control vector or a Sox17-construct to over-express Sox17. Scale bar = 50 μm (original panel) and 20 μm (enlarged panel). *n* = 6. OE, overexpression. **b** Co-localization coefficient for the fraction of Flag in CD31^+^ cells assesses the transgene expression in the endothelium. The Pearson correlation coefficient is significantly increased in Sox17-overexpressing mice compared to control mice. *n* = 6. **c** Western blot analysis and its quantification **d** showed a significant increase in the flag and Cyclin E1 expression in the pulmonary endothelial cells of mice with 3 days of Sox17 overexpression compared to vector mice. *n* = 3. **e** Quantification of BrdU^+^ nuclei in each field of 425 μm^2^ area in lung cryo-sections from vector-overexpressing and Sox17-overexpressing mice. *n* = 5 per group and 6 technical replicates per sample. Slides are co-stained with CD31-AF594, BrdU-AF488, and DAPI. Both groups show increased BrdU^+^ ECs at day 3 post-LPS as compared to baseline and the response was significantly greater in mice in which ECs overexpressed Sox 17. **f** Lung transvascular albumin permeability pre-LPS and post-LPS challenge in mice overexpressing endothelial Sox17 and control mice. *n* = 5. Mice overexpressing Sox17 in ECs showed significantly reduced vascular leakiness post-LPS when compared to control mice. **g** Survival curve of LPS challenge in control mice and mice over-expressing Sox17 in the endothelium. *n* = 11 per group. At this lethal dose of LPS (20 mg/kg), the death rate for control mice is 60% while for Sox17-overexpressed mice is 10%. **h** Model. LPS induces tissue hypoxia due to local oxygen depletion by infiltrating activated neutrophils, thereby stabilizing HIF-1α resulting in upregulation Sox17 expression and Sox17 mediated expression of Cyclin E1. This activates cell cycle re-entry and EC proliferation, and restoration of endothelial integrity. **P < 0.01 and ***P < 0.001. Data are shown as mean ± SEM. Analysis was performed using two-way ANOVA with Bonferroni post-tests for (**d**–**f**) and Log-rank (Mantel-Cox) test for (**g**)

To ensure that i.v. plasmid delivery using cationic liposomes induced expression in lung ECs^35–37^, we cloned a 2.5 kb *Cdh5* promoter region from mouse genomic DNA and used it to guide Flag-tagged Sox17 gene expression in a pCDNA3.0 vector because VE-cadherin is an endothelial-specific protein and its promoter thus specifically regulates endothelial-specific gene expression. Injection of the plasmid-liposome mixture i.v. in mice 3 h after LPS challenge induced expression as shown by Flag-tag and co-staining for the endothelial protein CD31 from day 3 onwards (Fig. 6a, b). Immunoblotting of lung ECs (Fig. 6c, d) showed significant increases in Flag and Cyclin E1 expression within 3 days of Sox17 overexpression as compared to vector control mice (Supplementary Fig. 9). Immunostaining of CD31 and BrdU (Supplementary Fig. 6E) showed significantly greater double positive lung ECs in mice overexpressing Sox 17 as compared to controls. EC proliferation increased 3-fold at day 3 post-LPS in mice overexpressing Sox17, which was coupled to restored lung endothelial barrier function, whereas barrier remained leaky in mice receiving control vector (Fig. 6f). Upregulation of Sox17 in lung ECs also significantly improved survival in mice as compared to controls (Fig. 6g).

## Discussion

We demonstrate using endothelial lineage tracing analysis in mice that endothelial cells which survive endotoxin-induced EC injury are themselves the primary source of ECs re-populating the vessel wall and restoring EC barrier integrity. Furthermore, we established the central role of HIF-1α signaling in activating the developmental transcription factor Sox17 in ECs, which induced EC proliferation through expression of Cyclin E1. These findings show the central role of the HIF-1α-Sox17-Cyclin E1 signaling axis in mediating EC regeneration following LPS-induced EC injury (Fig. 6h).

Multiple mechanisms of EC regeneration have been proposed, including circulating endothelial progenitor cells (EPCs)^45^, bone marrow c-kit^+^ cells^46^, and resident fibroblasts that convert to ECs^47^; however, the mechanisms of pulmonary EC regeneration in inflammatory lung injury have remained unclear^48,49^. Here, using mTmG-endo-*Scl*-CreERT2 mice in which native ECs are permanently labeled with EGFP and non-ECs are labeled with TdTomato, we carried out a lineage tracing study^29,50^ following inflammatory EC injury induced by the bacterial endotoxin LPS. We observed that EC injury resulted in severe EC loss, consistent with histological observations that lung EC is the predominant cell injured by LPS^51^. We tested various concentrations of LPS, and empirically arrived at 12 mg/kg LPS which induced maximal loss of ECs without causing lethality yet enabled quantitative assessment regeneration. EC population decreased sharply at day 1 post-LPS and progressively recovered within 7 days as evident by both EC lineage tracing in vivo and FACS analysis of ECs isolated from lungs. The loss of ECs seen reflected severe EC injury and denudation of lung endothelium characteristic of endotoxin-induced ALI^6^. EC loss was accompanied by increased lung vascular permeability to albumin within the first day, which returned to basal levels paralleling the restoration of EC coverage in the pulmonary vascular bed within 7 days post-LPS. To address the mechanism of EC recovery, we focused on the lung ECs and identified Sox17 as a key regulator of recovery. However, it remains unclear whether Sox17 also mediates EC regeneration in other vascular beds. A recent analysis of transcriptomic and signaling heterogeneity of ECs derived from distinct vascular beds^52^ suggests that gene regulatory networks and regenerative pathways can differ in distinct vascular beds.

The mTmG reporter mice used in the present study employed the endothelial-specific Cre reporter—endo-*Scl*-CreERT2 driven by a 5′ endothelial enhancer of stem cell leukemia (*Scl*) locus^29^. *Scl* expressed in hematopoietic cells is driven by the *Scl* 3′ enhancer whereas the 5′ enhancer modification used in the present study ensured EC specificity. This was confirmed by FACS analysis which showed that 95% of EGFP^+^ cells were indeed ECs. To confirm these findings, we also used another Cre model—the *Cdh5* CreERT2 genetic mouse^34^. Both of these models of EC-specific Sox17 deletion demonstrated the requisite role of Sox17 in mediating regeneration of ECs.

We found increased lung vascular permeability at day 1 post-LPS; however, there was no difference in the severity of the permeability response between *Sox17*^*EC−/−*^ and control *Sox17*^*fl/fl*^ mice. Lung vascular permeability measured in subsequent days during the recovery phase identified a critical role for EC Sox17 in regeneration of the endothelium. Importantly, *Sox17*^*EC−/−*^ mice exhibited persistent loss of endothelial barrier integrity indicative of failed EC regeneration program. This study thus demonstrated the requisite role of Sox17 in mediating EC regeneration.

As a member of the Sry-related high mobility group domain family F (SoxF) transcription factors^20^, Sox17 regulates endothelial and hematopoietic development during embryogenesis^21,23,24^. Its role in the adult has been studied in tumor development^26^, intracranial aneurysm formation^25^, and trans-differentiation of fibroblasts to ECs^27^. In the present study, we identified the central role of endogenous Sox17 in mediating EC regeneration following LPS induced injury by upregulating  Cyclin E1. These results mirror findings in quiescent adult hematopoietic stem cells (HSCs) showing that Sox17 over-expression gave rise to fetal-like HSCs with high self-renewal capacity^53^.

Sox7 and Sox18 are other members of the SoxF family that may play a complementary roles in vascular development^54–56^. During embryogenesis, Sox18 induces lymphatic endothelial cell commitment^54,56^. Sox7 and Sox18 also regulate venous-arterial vascular differentiation^57^ while Sox17 is expressed in arterial vessels and regulates their development^24^. Since expression of Sox7 and Sox18, unlike Sox17, did not increase during screening of transcription factors in regenerating ECs, their role in EC regeneration may not be primary. A recent study demonstrated upregulation of Sox7 and downregulation of Sox17 during vessel growth of high-grade glioma^58^ and Sox17 deletion in tumor vessels increased Sox7 expression^58^. Sox18 also appears to be involved in melanoma vascularization^59^. The opposite pattern of expression of Sox7 and Sox17 and the involvement of Sox18 in tumor vascularization suggests that SoxF family members may have different functions in ECs dependent on the niche, insult, or type of vascular beds.

ER71 (also known as ETV2) is an EC-specific developmental transcription factor regulating neovascularization in hind-limb ischemia in mice^31^. We did not detect any change in ER71 expression following LPS. It is possible that the mode of injury itself determines activation of the transcriptional regenerative program in ECs. As we showed, Sox17 is essential for regulating EC regeneration following endotoxin-induced EC injury whereas ER71 may be important in other forms of EC injury such as ischemia^31^.

The finding that transmigration and activation of neutrophils rapidly depletes tissue oxygen, and stabilizes HIF in surrounding cells^60^ prompted us to investigate tissue hypoxia and downstream HIF signaling pathways as a factor mediating EC regeneration. We observed in *Hif1a*^*EC−/−*^ mice that Sox17 expression in ECs required HIF-1α, and further we identified HIF-1α as the upstream transcriptional mechanism of Sox17 expression. HIF-1α is known to regulate EC proliferation by activation of VEGFR2 in tumor angiogenesis^61^ and Sox17 also regulates tumor angiogenesis through upregulating VEGFR2 expression^26^. We focused on Cyclin E1 as a downstream target of Sox17 responsible for EC proliferation and thus restoring EC coverage in the severe EC injury induced by LPS. Cyclin E1 plays an essential role in cell cycle regulation by inactivating APC^cdh1^ and transitioning cells into the proliferative phase^32^. We demonstrated that Sox17 induced EC proliferation through increasing the expression of Cyclin E1. We also identified Sox17 binding sites in the *CCNE1* promoter responsible for Cyclin E1 expression. Thus, LPS induced EC injury induced Sox17 expression and Sox17 in turn induced EC proliferation through the expression of Cyclin E1.

The concept that upregulation of HIF-1α expression or activity can used therapeutically to regenerate ECs is problematic. HIF-1α activation has multiple collateral effects because it regulates processes as diverse as tumor growth, angiogenesis, cell proliferation, and metabolism^20,61–63^. Based on our findings, a more tenable downstream target to enhance EC regeneration may be Sox17. We demonstrated that liposomal delivery of Sox17 in lung ECs augmented endothelial regeneration, accelerated endothelial barrier restoration, and reduced mortality. Thus, enhancing proliferation of ECs through upregulation of Sox17 may be useful strategy for restoring endothelial integrity and normalizing perfusion.

We relied on the use of i.v. injected liposomes containing *Sox17* cDNA to upregulate the expression of Sox17 in ECs^36,37,64^ of *Sox17*^*EC−/−*^ mice. The shortcoming of this approach is that Sox17 expression may not be confined to ECs. To obviate this concern, we cloned *Sox17* cDNA under the control of the EC specific *Cdh5* promoter^34^. Also previous studies have shown liposome formulation used leads to transgene overexpression in the lung endothelium and increased protein expression within 2–3 days of the plasmid injection^36,37^ (Supplementary Fig. 7). Additionally, our data also indicates that using the *Cdh5* promoter significantly increased transgene expression in the lung compared to all other organs (Supplementary Fig. 13), likely due to the high fraction of endothelial cells in the lungs. Functional assessment of Sox17 overexpression showed that lung EC expression of Sox17 promoted regeneration of ECs and restored endothelial permeability consistent with the time course of expression of Sox17 in ECs.

During liposome preparation, steps are typically employed to downsize the vesicles formed using either sonication or extrusion. These steps determine the polydispersity index (PDI), a measure of size distribution of the liposomes. PDI values can range from 0 for particles of uniform size to 1 where there is a broad size distribution. While nanoparticles synthesized from polymeric building blocks usually have PDI < 0.2 µm^65^, liposomes are typically more heterogeneous; e.g., cholesterol liposomes prepared using a single extrusion step have PDI of 0.4 µm^66^ whereas DDAB containing liposomes have PDI values between 0.13 and 0.48 µm depending on the exact liposome formulation^67^. An even wider range of PDI values can be found in the literature^68–70^. Thus, the method used to prepare liposomes influences the PDI value^66^. We observed that simply changing the syringe filter size from 0.45 to 0.22 µm resulted in a narrower size distribution and lowered PDI value from 0.3–0.4 µm to 0.2 µm (Supplementary Fig. 12). For clinical applications a low PDI is preferable as it suggests a more uniform product. While the PDI value of the liposomes in the present studies was sufficient to induce expression of Sox17 and effectively regenerate lung vessels, a limitation of our study is that we do not know the optimal liposome size for most efficient cDNA delivery to the pulmonary endothelium.

We showed that expression of Sox17 in ECs is directly regulated by HIF-1α activation. However, we cannot rule out the possibility that endotoxemia can activate multiple inflammatory pathways and release paracrine growth factors and cytokines, which may also contribute to the expression of Sox17. Wnt signaling is known to upregulate Sox17 expression^24,71^ and activation of the inflammatory transcription factor NF-κB triggers Wnt signaling^72–74^; thus, it is possible that such an inflammatory pathway can induce Sox17 expression parallel to the HIF-1α signaling pathway mediating Sox17 expression we have described.

In conclusion, the present study showed that induction of Sox17 expression through the activation of HIF-1α in ECs plays an essential role in regenerating ECs following endotoxin-induced EC injury. Sox17 functions by transcribing Cyclin E1 and activates proliferation of ECs, to re-anneal the injured endothelium. The HIF-1α-Sox17-Cyclin E1 signaling pathway functions as a critical mechanism of EC regeneration and may serve as a therapeutic approach for promoting the regeneration of the endothelium.

## Methods

### Generation of mutant mice

All animal experiments were conducted in accordance with NIH guidelines for the Care and Use of Laboratory Animals and were approved by the IACUC of the University of Illinois. C57BL/6 mice (Strain #027) were purchased from Charles River Laboratory. ROSA^mT/mG^ mice (Jackson Lab, Stock #007576) were crossed with endothelial *Scl*-CreERT2 mice^29^ (provided by Dr. Joachim Göthert). Mice were gauge fed with Tamoxifen (Sigma #T5648; 20 mg/ml in corn oil) for consecutive five days and then rested for four weeks to induce EC-specific membrane EGFP expression. Afterward, mice were challenged with sub-lethal LPS (Sigma #L2630) i.p. 12 mg/kg and then sacrificed at different time points for tissue harvest. *Sox17*^*fl/fl*^ mice (Jackson Lab, Stock #027712) were crossed with endothelial *Scl*-CreERT2 mice and *Cdh5*-CreERT2 (provided by Dr. Ralf Adams) mice^34^, respectively. Mice were gauge fed with Tamoxifen (20 mg/ml in corn oil) for consecutive five days and then rested for four weeks to induce EC-specific Sox17 protein deletion. Afterward, *Sox17*^*EC−/−*^ and control (*Sox17*^*fl/fl*^ Cre negative mice with tamoxifen) mice were challenged with sub-lethal LPS 8 mg/kg i.p. and then sacrificed at different time points for tissue harvest. *Hif1a*^*fl/fl*^ mice (Jackson Lab, Stock #007561) were crossed with Tie2-CreERT2 mice^75^ (Jackson Lab, Stock #2450312). Mice were gauge fed with Tamoxifen (20 mg/ml in corn oil) for consecutive five days and then rested for four weeks to induce EC-specific HIF-1α protein deletion. Afterwards, *Hif1a*^*EC−/−*^ and control (*Hif1a*^*fl/fl*^ Cre negative mice with tamoxifen) mice were challenged with sub-lethal LPS 12 mg/kg i.p. and then sacrificed at different time points for tissue harvest. All experiments were performed with 8–12-week-old animals using age-matched and sex-matched groups. All animals were on a C57BL/6 background. The mice were maintained in a pathogen-free environment in University of Illinois at Chicago Animal Care Facility (Biological Resource Center). Mice were randomly assigned to treatment arms with approximately equivalent numbers in each group. No blinding was done for animal experiments. The present studies addressed mechanisms of lung endothelial repair in a model of ALI/Acute Respiratory Distress Syndrome (ARDS) which are typically complications of severe systemic infections (e.g., endotoxemia). Endotoxemia was induced by systemic LPS (i.p.) which is routinely used in experimental lung injury models. This model also mirrors lung injury seen with polymicrobial sepsis^76^.

### 2-photon microscopy

The tissue live imaging experiment followed a previously described protocol^77^. Generally, the mice were sacrificed, and the lung was flushed with Phosphate-buffered saline (PBS, Sigma #P3813). Then 1 ml 37 °C 2% low-melting-temperature agarose was slowly instilled through the trachea into the lung. Once the lungs were fully inflated, we let the lungs cool by pouring 4 °C PBS over the lung and removed the lungs from the mice. We sectioned the largest lobe of the lung into 300-μm slices and placed the slices on plastic coverslips in the imaging chamber. Images were taken with a Prairie Technologies Ultima In Vivo Multiphoton Microscopy System (Bruker) and analyzed by Imaris software (Bitplane).

### Mouse cell isolation

Flushed mouse lungs were  minced and digested with 5 ml Type 1 collagenase I (2 mg/ml in PBS) at 37 °C water bath for 1 h. Mixtures were titrated with #18 needles and then pipetted through a 40 μm disposable cell strainer. After centrifuging at 300 × *g* for 5 min and washing with PBS, the isolated cells were treated with red blood cell lysis buffer (eBioscience) for 5 min on ice to lyse red blood cells.

### Flow cytometry

After isolation, remaining cells were incubated with anti-mouse CD16/CD32 (1:50, BD Pharmingen #553142) to block endogenous Fc for 10 min on ice. After this, cells were stained with antibodies including CD45-EF450 (1:2000, eBioscience #48–0451–82) and CD31-APC (1:100, eBioscience #17–0311–82) for 45 min at 4 °C. After washing, the cells were resuspended in 500 μl buffer and analyzed on an LSRFortessa (BD Pharmingen) cell analyzer. Obtained data were analyzed by Summit software (Beckman Coulter).

### Mouse EC pull down assay

After isolation, cells were incubated with 5 µg CD31 antibody (10 µl antibody in 1 ml buffer, BD #553370) at 4 °C for 20 min with gentle tilting and rotation. After washing, cells were then incubated with pre-washed Dynabeads (25 µl beads in 1 ml buffer, Invitrogen #11035) at 4 °C for 25 min with gentle tilting and rotation. ECs were harvested by magnetic separation.

### RNA isolation and qPCR

RNA was extracted from the harvested cells using TRIzol™ Reagent (ThermoFisher #15596026) according to the manufacturer’s protocol. Then RNA was quantified by Nanodrop 1000 (ThermoFisher) and reverse transcribed into cDNA using High-Capacity cDNA Reverse Transcription Kit (ThermoFisher #4368814). FastStart Universal SYBR Green Master (ThermoFisher #4913850001) was used for relative quantification of cDNA on the ViiA 7 Real-Time PCR System (ThermoFisher) (Primer information included in Supplementary Table 2).

### qPCR heatmap

The qPCR relative fold change values for a set of genes were plotted using the heatmap.2 function in the R “gplots” package. Upregulation of genes is shown in red, white, and green, respectively. Red corresponds to minimal increase while green corresponds to dramatic increase relative to the untreated control.

### Immunoblotting

Harvested cells or tissues were lysed in RIPA buffer (Sigma #R0278) with protease (Sigma #P8340) and phosphatase inhibitor (Sigma #P5726 and #P0044). Protein concentration was measured using DC™ Protein Assay Kit II (Bio-Rad #5000112) in Epoch Microplate Spectrophotometer (BioTek). Immunoblotting was performed with Sox17 (1:250, Origene #AM32707PU-N for mouse Sox17; 1:200, R&D #AF1924 for human Sox17), HIF-1α (1:200, Cayman Chemical #10006421), HIF-2α (1:500, Novus Biologicals #NB100–122), Flag M2 (1:1000, Sigma #F1804), Cyclin E1 (1:500, R&D #AF6810) primary antibody using Bio-Rad Protein Electrophoresis and Blotting system. Sample loading was confirmed by GAPDH expression (1:5000, Proteintech #60004). Western blot pictures were taken using ImageQuant LAS 4000 (GE Healthcare). Uncropped versions of all Western blots are available as a Source Data file.

### Cell culture

HLMVECs (Lonza #CC-2527) were maintained in culture in a humidified 5% CO_2_ atmosphere at 37 °C in complete Clonetics EGM-2MV BulletKit medium (Lonza #CC-3202) and 15% fetal bovine serum (Atlanta Biologicals). Cells were thawed at passage 3 and transduced at the same passage. For hypoxic signaling activation studies, HLMVECs were treated with the HIF1α activator DMOG (Calbiochem #400091). 293T cells (ATCC #CRL-3216; Clontech #632180) were maintained in culture in a humidified 5% CO_2_ atmosphere at 37 °C in DMEM medium (Gibco #11995–065) with 10% fetal bovine serum (Atlanta Biologicals).

### CRISPR/Cas9 studies

Guide RNA targeting human HIF-1α and HIF-2α gene for Cas9-mediated CRISPR disruption were identified using online design software at http://crispr.mit.edu. The two targeting sequences for HIF-1α were: 5′-TTCTTTACTTCGCCGAGATC-3′ and 5′-CCTCACACGCAAATAGCTGA-3′. The two targeting sequences for HIF-2α were: 5′-GCTGATTGCCAGTCGCATGA-3′ and 5′-CAAGGCCTCCATCATGCGAC-3. The transduction of lentiviral sgRNA and adenoviral Cas9 followed a previous protocol^42^. Generally, DNA oligos were synthesized by IDT and the overlapping PCR products were cloned into pLX-single sgRNA lentiviral vector (a gift from Eric Lander and David Sabatini^78^ and obtained through Addgene as plasmid #50662). Lentivirus was prepared by co-transfection of lentiviral plasmids with psPAX2 (produced by Didier Trono and obtained through Addgene as plasmid #12260) and pMD2.G (produced by Didier Trono and obtained through Addgene as plasmid #12259) packaging plasmids into 90% confluent human 293T cells using Lipofectamine 2000 as per manufacturer’s protocol. Lentiviral supernatant was collected at 48 and 72 h post-transfection, concentrated by Lenti-X concentrator (Clontech #631232) as per manufacturer’s protocol, and used to transduce HLMVECs at a Multiplicity of infection (MOI) of 10 (measured by Global UltraRapid Lentiviral Titer Kit, System Biosciences #LV961A-1) in the presence of 8 μg/ml polybrene (Sigma #TR-1003). At 8 h later, the EGFP-tagged Cas9 adenovirus (Ad-GFP-Cas9, Vector Biolabs #1901) was also added at a MOI of 10 to transduce HLMVECs. After 2–3 days when HLMVECs reached confluency, the cells were trypsinized and split 1:3 and received a second batch of sgRNA lentivirus and EGFP-Cas9 adenovirus. Two to three days later, the cells were ready for experimental studies.

### BrdU assay of EC proliferation

Mice were injected i.p. with BrdU (Sigma #B5002) 150 mg/kg 14 h before sacrifice. After the mice being sacrificed, mice lung was flushed with PBS, infused with 70% OCT intratracheally and then excised in the cassette and put on dry ice. After storage at  −80 °C for at least 48 h, mouse lungs were sectioned into 5 µm frozen sections.

### Immunofluorescence and confocal microscopy

The frozen sections were fixed with 4% paraformaldehyde (Sigma #P6148) and permeabilized with 0.25% Triton X-100 (Fisher Scientific #BP151–100). After a wash with PBS/Tween-20 (Fisher Scientific #BP337–100), slides were treated with 3 M hydrochloric acid (Fisher Scientific #A144S-500) for 10 min to open the nucleus structure if needed. Slides were blocked with 10% donkey serum (Sigma #D9663) in 2% BSA (Sigma) in PBS/Tw-20 for 1 h at room temperature. Afterwards, slides were probed with primary antibodies (CD31 1:25, BD #550274; BrdU-APC 1:50, BD; Alexa Fluor 488 Mouse anti-BrdU 1:10, BD #558599; Flag M2 1:500, Sigma #F1804) and incubated overnight at 4 °C. The next day, slides were washed and incubated with the fluorescence-conjugated secondary antibody (AF488 donkey anti-rat 1:300, Invitrogen #A-21208; AF594 donkey anti-mouse 1:300, Invitrogen #A-21203; AF594 donkey anti-rat 1:300, Invitrogen #A-21209). Images were taken with a confocal microscope LSM880 (Zeiss) and analyzed by Zen software (Zeiss).

### Sox17 binding sites in *CCNE1* promoter

We identified human *CCNE1* promoter region using the UCSC genome browser. For the prediction of human Sox17 binding sites in the promoter of *CCNE1*, we searched for the presence of ATTGT motifs in the JASPAR database. The binding sites were found to be at −1090 ~ −1082 bp, −1335 ~ −1327 bp, −2022 ~ −2014 bp, and −2092 ~ −2084 bp upstream of the *CCNE1* ATG start site.

### Identification of HREs in *Sox17* promoter

We performed a sequence alignment of the human *SOX17* promoter region using ClustalW2. For the prediction of human HIF-1α binding sites in the promoter of *SOX17*, we searched for the presence of RCGTG motifs. In addition, we only considered RCGTG motifs that were conserved in, at least, four species, including the mouse. The HREs were found to be at −294 ~ −285 bp, −318 ~ −311 bp, and −338 ~ −330 bp upstream of the *SOX17* ATG start site.

### Chromatin-immunoprecipitation assay

The Ch-IP protocol is based on the protocol of Bryan Dynlacht and Richard Young laboratories. For the Sox17—*CCNE1* experiment, HLMVECs transduced with pMXs-ms-Sox17 (a gift from S. Yamanaka^79^, Addgene plasmid #50781) were crosslinked by using 1% formaldehyde (Fisher Scientific #F79–1), washed three times with cold PBS, and resuspended in cell lysis buffer. The nuclei portion was resuspended in nuclear lysis buffer and sonicated to break down the genomic DNA using S220 Focused-ultrasonicator (Covaris). After centrifugation, the supernatant was immunoprecipitated with 5 μg anti-Sox17 (R&D #AF1924) or an equal amount of goat IgG (Santa Cruz #sc-2028). The DNA obtained from the IP was amplified by qPCR with primers specifically recognizing different binding sites in *CCNE1* promoters (Primer information included in Supplementary Table 2). For the HIF-1α-*SOX17* experiment, HLMVECs cultured in normoxic or 1% O_2_ hypoxic conditions were processed as above and then immunoprecipitated with 5 μg anti-HIF1α (Santa Cruz #sc-10790) or an equal amount of rabbit IgG (Santa Cruz #sc-2027). The DNA obtained from the IP was amplified by qPCR with primers specifically recognizing different HRE sites in *SOX17* promoters (Primer information included in Supplementary Table 2).

### Myeloperoxidase activity

Mice lungs were flushed with PBS and homogenized in RIPA buffer (Sigma #R0278). 50 μg extracted sample was mixed with 1 ml of 50 mM phosphate buffer (Sigma #S3264 and #S3139) containing 0.167 mg/ml of o-Dianisidine dihydrochloride (Sigma #D3252) and 0.0005% H_2_O_2_ (Sigma #216763). The reaction proceeded for 3–5 min at room temperature, and then was stopped by adding 3 M Hydrochloric acid (Sigma #H1758). Absorption was determined by OD460 using UV/vis spectrophotometer (Beckman #DU530) and normalized by protein concentration measured using DC™ Protein Assay Kit II (Bio-Rad #5000112) in Epoch Microplate Spectrophotometer (BioTek).

### Evans blue-albumin tracer measurement of pulmonary transvascular permeability

Evans blue–albumin (EBA) (40 mg/ml BSA with 1% Evans blue dye Sigma #E2129) was injected into the right jugular vein of the mice (anesthetized by ketamine-xylazine mix approved from pharmacy) at a dose of 8 µl per mouse body weight gram and allowed to circulate in the blood vessels for 45 min. Intravascular Evans blue was washed by PBS perfusion from the right ventricle for 2 min. Mouse lungs were excised, homogenized in 1 ml PBS, and extracted in 2 ml Formamide (Fisher Chemical #F84–1) overnight at 60 °C. Evans blue content was determined by OD620 using UV/vis spectrophotometer (Beckman #DU530) of the formamide extract and normalized by body weight. The body weights of mice for the Evans blue experiments are provided in Supplementary Table 1.

### Plasmid constructs

The 2.5 kb *Cdh5* promoter was cloned from mouse genomic DNA and then inserted into the pCDNA 3.0 vector. The original CMV promoter was destroyed. Then the mouse Sox17 protein coding region was amplified from pMXs-ms-Sox17 plasmid and inserted into the pCDNA-*Cdh5* vector. A flag tag (GACTACAAAGACGATGACGACAAG) was added to the N-terminus of the *Sox17* coding region. For the liposome-gene complex biodistribution experiment, the EGFP protein coding region was amplified from a pWPXL plasmid (a gift from Didier Trono, Addgene plasmid #12257) and inserted into the pCDNA-*Cdh5* vector. Full-length human *SOX17* and *CCNE1* promoter plasmids (GenoCopeia #HPRM50295-PG04 and #CS-HPRM38973-PG04–01) and the truncation mutants (PCR amplified) were subcloned into a pGL3-basic vector (Promega #E1751). The HIF-1α constitutively active mutant HA-HIF1alpha P402A/P564A-pBabe-puro was a gift from William Kaelin (Addgene plasmid #19005)^80^. Control pRL-TK plasmid was purchased from Promega (#2241).

### Luciferase reporter assay

293T cells were seeded in 24-well cell culture plates and transfected with the indicated plasmids using Lipofectamine 2000 (Invitrogen #11668). One day after transfection, cells were lysed, and luciferase activity was measured using the dual-luciferase reporter assay system (Promega #E1910) on a GloMax luminometer (Promaga #SA3030). Relative luciferase units were determined by dividing the luciferase activity of the Firefly target pGL3 reporters by that of the pRL-TK Renilla luciferase control reporter.

### Liposome gene delivery in lung ECs

Dimethyldioctadecylammonium bromide (Sigma #D2779) was dissolved in Chloroform (Fisher Chemical #C606SK-1) in a 1:1 molar ratio with Cholesterol (Millipore #228111). Chloroform was evaporated in a Rotavapor (Buchi #R-300) at 37 °C, 100 rpm for 20 min. Afterwards, the dried lipid film was resuspended in 5% dextrose (Fisher Scientific #D16–500) in water and then sonicated in Branson 2510 Ultrasonic Cleaner (Sigma #Z244910) for 20 min, followed by 0.45 μm filtration. The mice were anesthetized with isoflurane (Piramal Critical Care) and retro-orbitally i.v. injected with the liposome-DNA complex at a dose of 50 μg DNA/100 μg liposome per mouse. Size (hydrodynamic diameter) was 234.3 ± 8.3 nm for liposome only and 240.1 ± 3.4 for liposome-DNA complex (as shown in Supplementary Fig. 10). Polydispersity Index was 0.400 ± 0.027 for liposome-Vector complex and 0.414 ± 0.009 for liposome-Sox17 complex (as shown in Supplementary Fig. 12). The surface charge (Zeta Potential) was 39.8 ± 0.2 mV for liposome only and 34.8 ± 0.2 mV for liposome-DNA complex (as shown in Supplementary Fig. 11). *n* = 6. Supplementary Fig. 13 shows the organ biodistribution of the transgene expression delivered by liposomes, where lung is the organ with highest plasmid gene expression. *n* = 4. All these parameters were measured by dynamic light scattering (Zetasizer Nano ZS, Malvern). Serum was first ultracentrifuged for 2 h at 140,000 × *g* (Optima TLX Ultracentrifuge, Beckman Coulter) to remove extracellular vesicles that might interfere with size measurements. We used liposomes (DDAB/cholesterol) with a high charge ratio of + 4.5 (+fatty acid/-DNA). The DNA complex was cloned with Flag-tagged *Sox17* coding region following the EC-specific mouse *Cdh5* promoter to ensure EC transgene expression.

### Statistics

Immunoblot bands were analyzed for optical density using ImageJ (NIH) software. Quantification of replicate experiments is presented as the mean ± SEM. The student *t*-test, one-way and two-way ANOVA with Bonferroni post-tests were used to determine statistical significance, with a *P* value threshold of less than 0.05. Significance levels are indicated in the figures as *P < 0.05, **P < 0.01, and ***P < 0.001. Based on our experience, we expect changes in the gene/protein expression and function measurements to be detected with 3 mice per group, so the effect size was determined as *n* = 3 or *n* > 3. The variance between the groups that are being statistically compared was similar.

### Reporting summary

Further information on research design is available in the Nature Research Reporting Summary linked to this article.

## Supplementary information

Supplementary Information

Description of Additional Supplementary Files

Supplementary Movie 1

Supplementary Movie 2

Reporting Summary

Source Data

## Data Availability

The authors declare that all relevant data supporting the findings of this study are available within the paper and its supplementary information files or from the authors upon reasonable request. Please contact Asrar B. Malik (abmalik@uic.edu) for any inquiries.
