# Supplementary material for: Sox17 is required for endothelial regeneration following inflammation-induced vascular injury
Source: Nat Commun. 2019 May 9;10:2126. doi: 10.1038/s41467-019-10134-y (PMC6509327; doi:10.1038/s41467-019-10134-y)
Supplement: Supplementary file 1 — Supplementary Information [file 41467_2019_10134_MOESM1_ESM.pdf]

Supplementary Figure 1

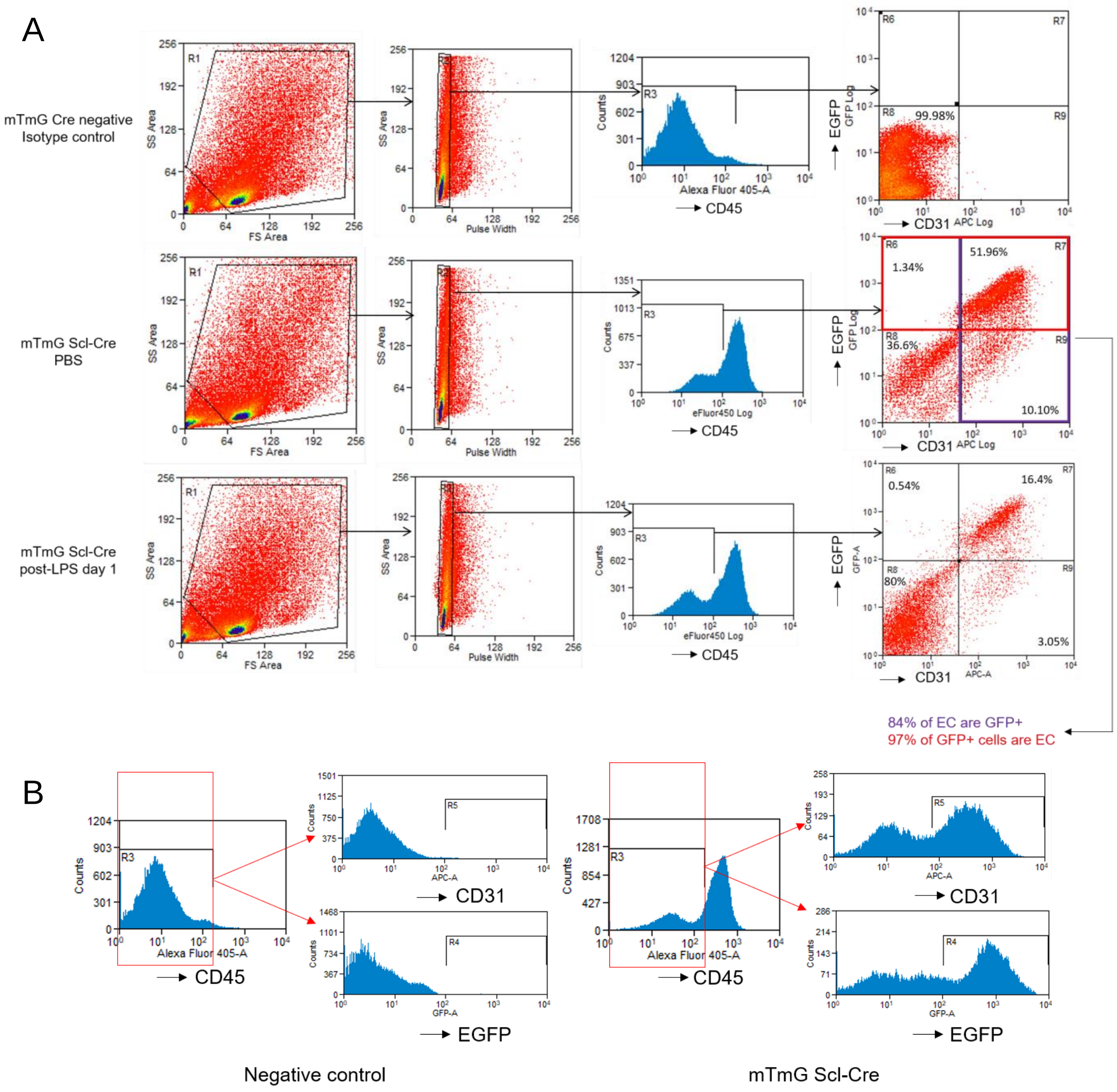

Supplementary Figure 1. Gating strategies and lineage tracing analysis showing high labeling efficiency and specificity. (**A**) Flow cytometry analysis of EGFP+ cell and CD31+ cell distribution in the whole lung population in Endo-Scl-Cre positive and negative mice pre- and post- LPS. We observed that 84% CD31+ ECs are EGFP+, suggesting high labeling efficiency. We observed that 97% EGFP+ cells are CD31+, showing that the transgenic mice are EC-specific. In the first gating R1, platelets are excluded based on the size. The analyzed population is also CD45 negative. Therefore, the identified CD31+ cell population is specific to endothelial cells. **(A)** and (**B**) Gating strategy to for CD31+ or EGFP+ cells among mice lung cell population in flow cytometry analysis for quantification in Figure 1D 1E and 3G.

Supplementary Figure 2

A

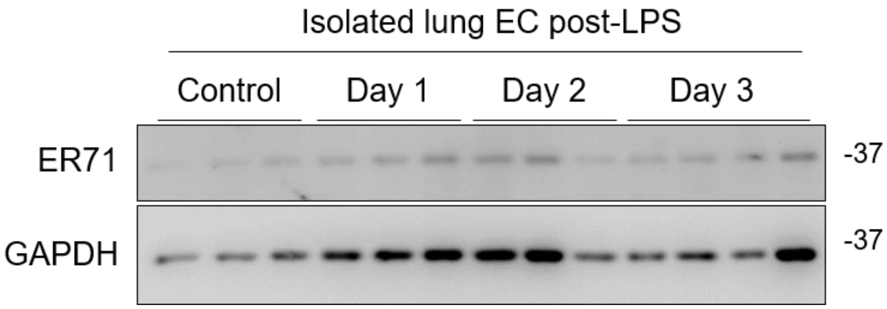

B

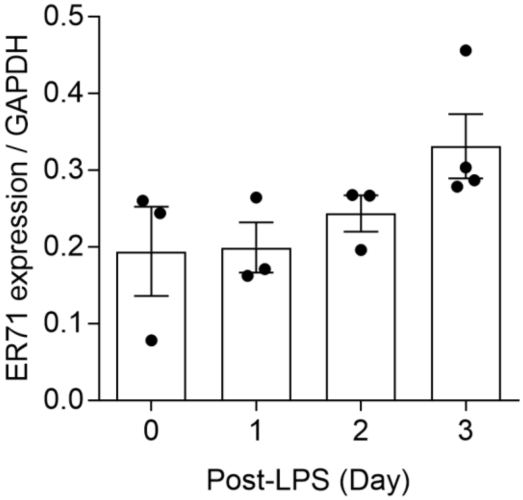

Supplementary Figure 2. ER71 expression does not change following LPS-induced acute lung injury. (A) Western blot analysis in freshly isolated ECs from wild-type mice and their quantification (B) show no significant change in Sox17 protein expression following EC injury compared to baseline. n=3. Data are shown as mean ± SEM. Analysis is performed using one-way ANOVA.

Supplementary Figure 3

Human *CCNE1* promoter sequence with Sox17 binding sites highlighted

1 GGTACTGTAC AGGAGTCAGC TACATTATCT CAATCTT**CAC AATGAT**CCTT

51 GGGAGTAGAT AGTTGCCATC TGTGAAACAG GATAATTGTG GCATCAACAT

101 GAATAGG**ATT ATTGTG**AAGT TTAATTGAGA TGATACTTTG AAGGACTTAG

151 CCCAGTGCTG CCATTTTGGA AACACTGTTT ATGATACCTG TTACTGGTGA

201 TTCCTAACGG GCACAGGATG GGGGGTTCAG GTGCCTGGGT CCCTGCTGAG

251 CAAGTAGCCC AGCAGCCTTG GCCTCAGAGC ACCTGGGGGA ATCATGAATT

301 TGTGAGCCTC TTTGATAAGA CCCTGGCTCT AACTTCAAGA AAACGGTCAC

351 TAGGGGAGGG TGAGGGACAG GACTGAGACT TTAGCTTAAG AAGGGCCCTG

401 GGAGCATTCC AGAGCCTTCT TTTACGCACA TCTTCCTGAA GCTGAGTACA

451 GAGGGTACTC TGAGGTGATG ACTGTTCCTT ACCTGTCTCT CTCATTAGCC

501 GGTAAGCCCT GCAAGGGCTT GGCCAGTGCC TGTCTTTCAA ATGTTCATTC

551 AGAAACAATT TAAGTGTCTC CCTTGGTCCA GGCACTATGC CAAGAACTGA

601 CAGATACAGC AGTGAGCAAG ATGGGCAAGG TGGGGAGAGA GACAAGAAAC

651 AGAGAGGCAC CAAGACTGTG TAGGGGCTGG GCTCCCAGCA CTTTGGGAGG

701 CCAAGGCGGG AGGATCGCTT GAGCTCATGG GTTTGAGACC AGCCTGAGCA

751 ACATAGCAAG ACCCTATCCC TACCTGCCAC CCCCCACCCC CCGC**CACAAT**

801 **AAA**AAATACA AGTCTTGAGG GAGAAAGGAC AGAGGAGCTG TGGAGAGGAG

851 GCCTGAGGTC TGAGGGTGGG GGATCAGGGT CAGGTCCTGT GGAGCCTGTA

901 GCCTAGGACA CGGAGTGTGG ATTTGACCCT TATGCGAAAC AACTGGAAGG

951 CTTTTTGTTT CTTTTTCTGT AGAAATGGGG GTCTCACTGT TACCCAGGCT

1001 GGTCTCGAAC ACCCCAAGGG ATCAGCCGTC TCGGCCTCC**C ACAATGCT**GG

1051 GATTAAAGGC GTGAGCCACC GCGCCCGGCC TCAACTGGAA GGCTTTAAGT

1101 GAGAGATGGG GTGCAAGGGA ATCCCAGAGT CAGAAAGGTC TTCAGAGAGC

1151 CAGGAAGGGC TTGCGGGGGA GGGGCGCATA TGGAAGGGGC GCATGGAAGG

1201 AACTCACAGA TTCCTTGAAT GAATGAATGA ACGACCCAAT GCACTGACGG

1251 ATGAATGGAC AGGCGGCCAG GAATAGCAGC CGGCCCCCAG GGAGCCCCAG

1301 ACCCCGCGGC CTGAAGCCTT GGTTCTAGGC CAAGGCACAG GCGCGGTGAC

1351 CTTGGGGATG TCCCCGCCCA GGACTCAGGG CCCGGAACTC GGCGTCTCGG

1401 GGGCGGGGAG GGCGTGCCTG GCGGGACAGC GCGCGCGGAG GAACGGCGGG

1451 CGGTGCTCCT CGGGTAGGCC CCCCACACAT CCCCTTGGCT CAGCCCTGCC

1501 GGGGCCCGAA CCCGCGCCGC CCGCCGTGTT TACATTCCAC CCGCGCCAGC

1551 CACGCGGCTT TTTGCCGCTC CAGCGCCGCT CGGCCCCGCC CCCGGCGCCC

1601 GCGGCCCGCC CCTCGCCGCC GCGCGCCAGA CTTCTCCCGC GTCCCGCCCG

1651 CCGCCCCGCC CCGCGTCCCG CGCCCCGCGC CCCGCGCCCG GCCCTCGGCG

1701 CGCAGGCCCT GTCACTTGGC CCCGCCCTGT CCGCCGGCCC CGCCCCTGAT

1751 TCCCCGTCCC TGCGCCTCGC GGGCCGGCGC CGTGGAGGGG CGGGTCCGGG

1801 GGCGGGGCGA GGGACGGGGC GGGACGGGCT CTGGGTCCCG CGCGGCCGCT

1851 GAGGGGCTGG GAGCCGCGGC GGGGCGGTGC GAGGGCGGGC CGGGGCCGGT

1901 TCCGCGCGCA GGGATTTTAA ATGTCCCGCT CTGAGCCGGG CGCAGGAGCA

1951 GCCGGCGCGG CCGCCAGCGC GGTGTAGGGG GCAGGCGCGG ATCCCGCCAC

2001 CGCCGCGCGC TCGGCCCGCC GACTCCCGGC GCCGCCGCCG CCACTGCCGT

2051 CGCCGCCGCC GCCTGCCGGG ACTGGAGCGC GCCGTCCGCC GCGGACAAGA

2101 CCCTGGCCTC AGGCCGGAGC AGCCCCATC**A TG**CCGAGGGA GCGCAGGGAG

Supplementary Figure 3. Human Cyclin E1 promoter sequence. Human Cyclin E1 promoter sequence 2000 bp upstream of ATG site, with Sox17 binding sites highlighted.

Supplementary Figure 4

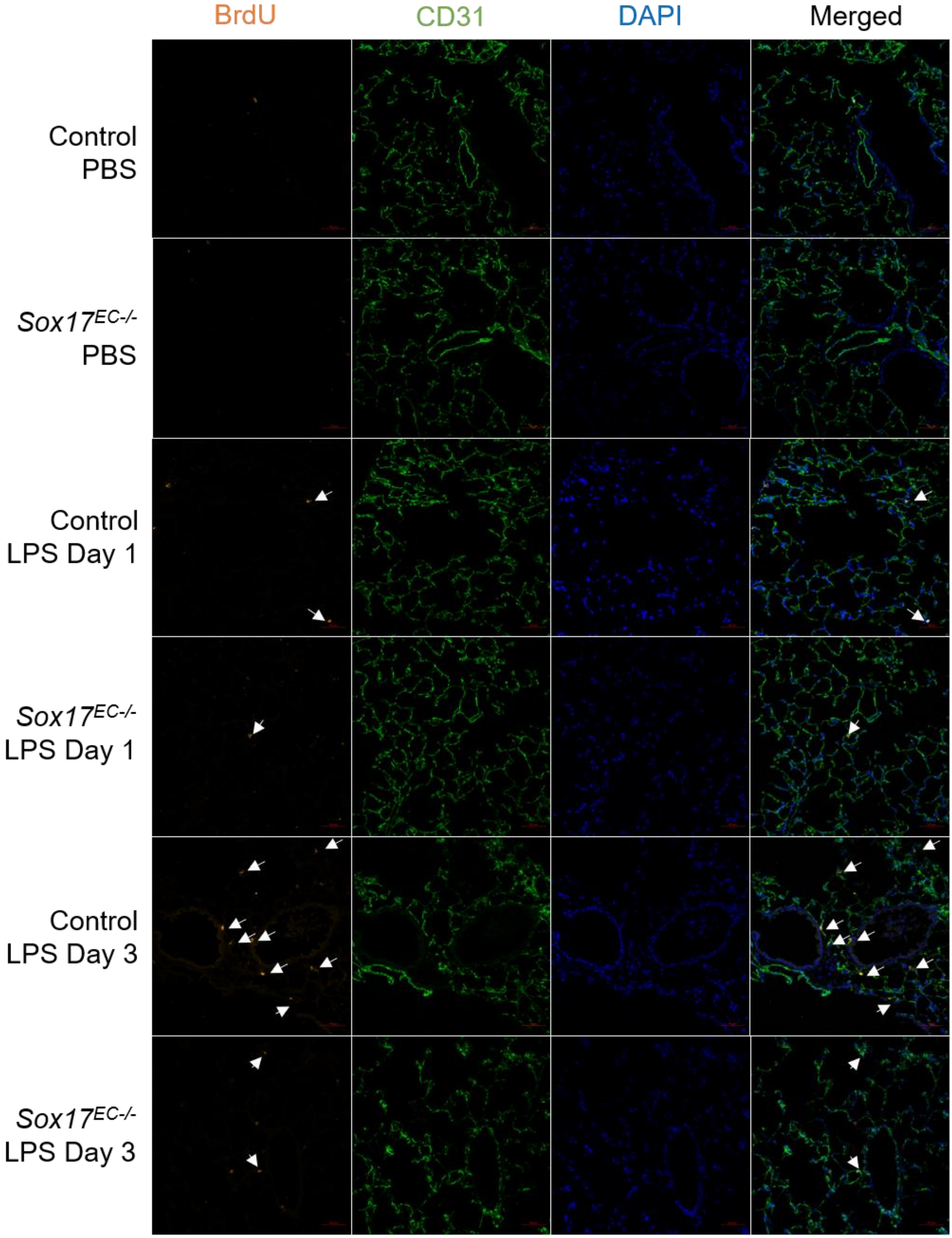

Supplementary Figure 4. *Sox17*$^{EC-/-}$ mice showing reduced EC proliferation following EC injury. BrdU+ nuclei staining with CD31 and DAPI co-staining in lung cryo-sections from wildtype and *Sox17*$^{EC-/-}$ mice. At day 3 post-LPS, the control group showed a significantly greater number of BrdU+ ECs compared to baseline. However, *Sox17*$^{EC-/-}$ mice showed markedly reduced BrdU+ ECs, indicating reduced EC proliferation. Arrows indicate BrdU+ EC nuclei.

Supplementary Figure 5

A Human *SOX17* promoter sequence with HREs highlighted

```
1   GGGAGGGGCAAGGGGCGGGCGTGGGGTTGGACTGGGACGTGGGACTCGGA
51  CCACGGCCTGGGCGTGGGCCTAACGACGCGGACCGGCCCGCCCTCGCCG
101 CTCCATTGGCCACATCTGTGCAGAAAGGCCCCGCGGCCCAGGGGCGCCC
151 GCAGTGTCACTAGGCCGGCTGGGGGCCCTGGGTACGCTGTAGACCAGACC
201 GCGACAGGCCAGAACACGGGCGGCGGCTTCGGGCCGGGAGACCCGCGCAG
251 CCCTCGGGGCATCTCAGTGCCTCACTCCCCACCCCCTCCCCCGGGTCGGG
301 GGAGGCGGCGCGTCCGGCGGAGGGTTGAGGGGAGCGGGGCAGGCCTGGAG
351 CGCCATGAGCAGCCCGGATGCGGGATACGCCAGTGACGACCAGAGCCAGA
```

B

```
                **
Human      CGGCCTGGGCGTGG-GCCTAACGACGCGGG---ACCGGCCCGC-CCTCGCCGCT-CCATTGGCCACATCTG
Mouse      CTGGCTGGGCGGGG-GCCTAAGGACTAGGG---TCCGGTCCGC-CCCCGCCTCG-CCATTGGCCGCGTCCA
Rat        CCGGCTGGGCGGGG-GCCTAAGGACTAGGG---TCCGGCCCGC-CCCCGCCGCG-CCATTGGCCAGGTCCA
Baboon     CGGCCTGGGCGTGG-GCCTAACTACGCGGG---ACCGGCCCGC-CCTCGCCGCT-CCATTGGCCACATCTG
Rhesus     CGGCCTGGGCGTGG-GCCTAACTACGCGGG---ACCGGCCCGC-CCTCGCCGCT-CCATTGGCCACATCTG
Orangutan  CGGCCTGGGCGTGG-GCCTAACGACGCGGG---ACCGGCCCGC-CCTCGCCGCT-CCATTGGCCACATCTG
Gorilla    CGGCCTGGGCGTGG-GCCTAACGACGCGGG---ACCGGCCCGC-CCTCGCCGCT-CCATTGGCCACATCTG
Dog        CCGGCGGGGCGTGG-GCCTAAGGACGCCCG----CCGGCCCGC-CCCCGCCACT-CCATTGGCCGCAGCAG
Horse      GTGGCTGGGCGTGG-ACCTCAGGACGCCCT---CCCGGCCCGC-CCCCGCCGCT-CCATTGGCCACGTCGG
Elephant   CGGGCTGGGCGTGG-GCCTAAGGACGCCCG----CCGGCACGC-CCCCGGCGCT-CCATTGGCCACCGCGG
Dolphin    CGAGCTGGGCGTGG-GCCTAGCGACGCCGG---CCCGGCCCGC-CCCCGTCGCT-CTATTGGCCACGTCTG
Cow        CGGGCTGGGCGTGG-GCCTAACGTAGCCGG----CCGGCCCGC-CCCCGCCGCT-CCATTGGCCGGGTCTG
Rabbit     CGGGCTGGGCGTGG-GCCTAACGCCTTGGG--CCCCGGCCCGC-CCCCGCTGCT-CCATTGGTCACATCTG
```

C

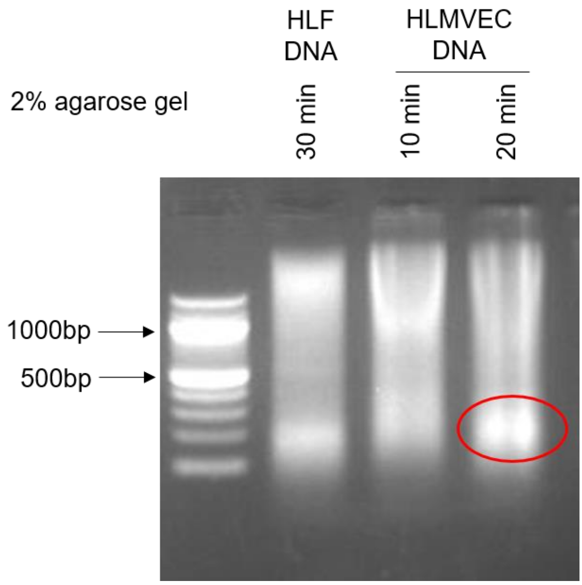

Supplementary Figure 5. Human Sox17 promoter sequence. (A) Human Sox17 promoter sequence 300 bp upstream of ATG site, with HREs highlighted. (B) One HRE in Sox17 promoter is conserved through species examined. (C) HLMVEC DNA shows a band at ~200 bp in agarose gel for Ch-IP after 20 min of sonication.

Supplementary Figure 6

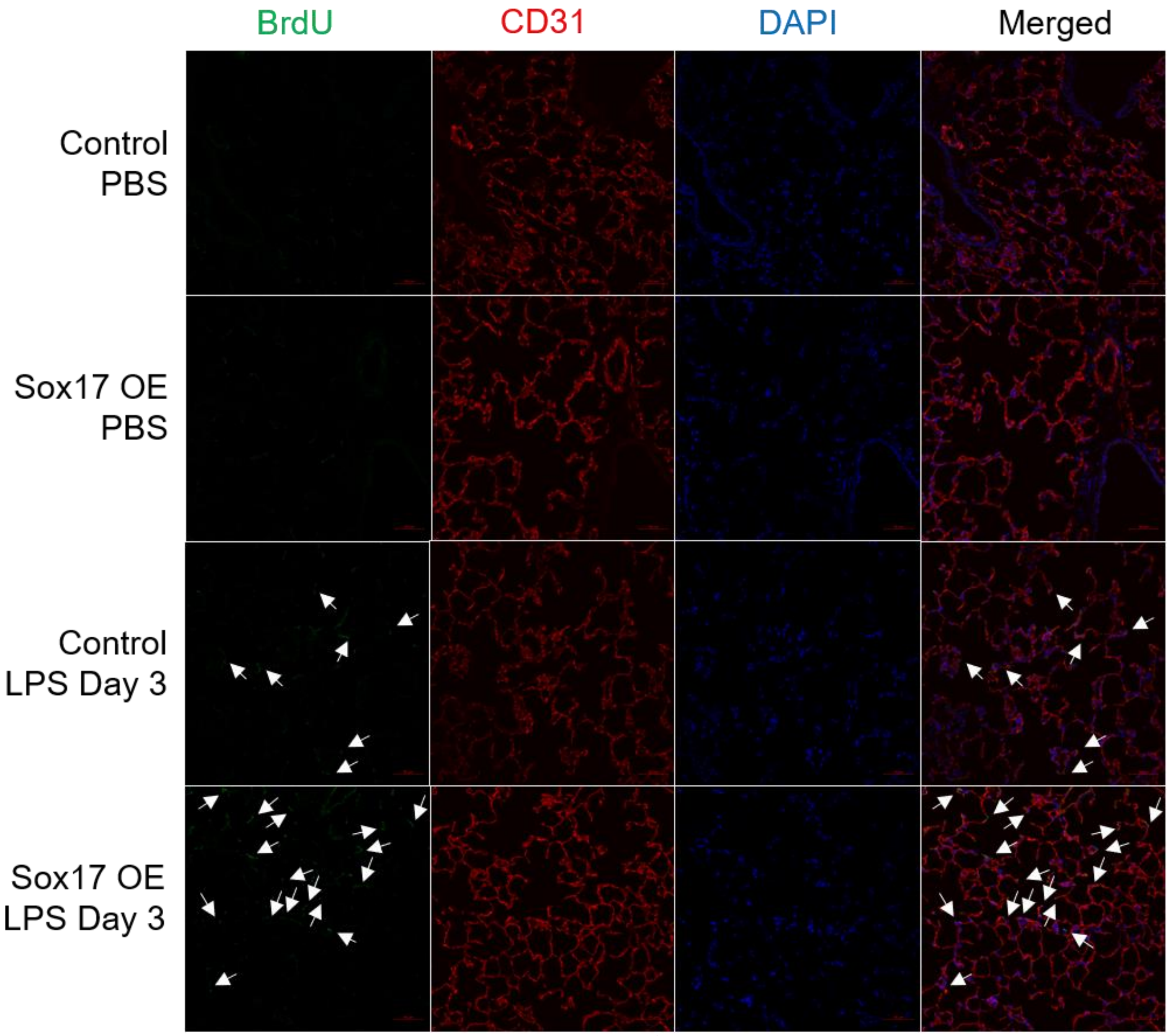

Supplementary Figure 6. Endothelial Sox17-overexpression in mice increases EC proliferation following injury. BrdU+ nuclei staining with CD31 and DAPI co-staining in lung cryo-sections from wildtype and Sox17-overexpressing mice. Both groups show increased BrdU+ ECs at day 3 post-LPS as compared to baseline and the response is significantly greater in mice in which ECs overexpressed Sox 17 than controls. Arrows indicates BrdU+ EC nuclei.

Supplementary Figure 7

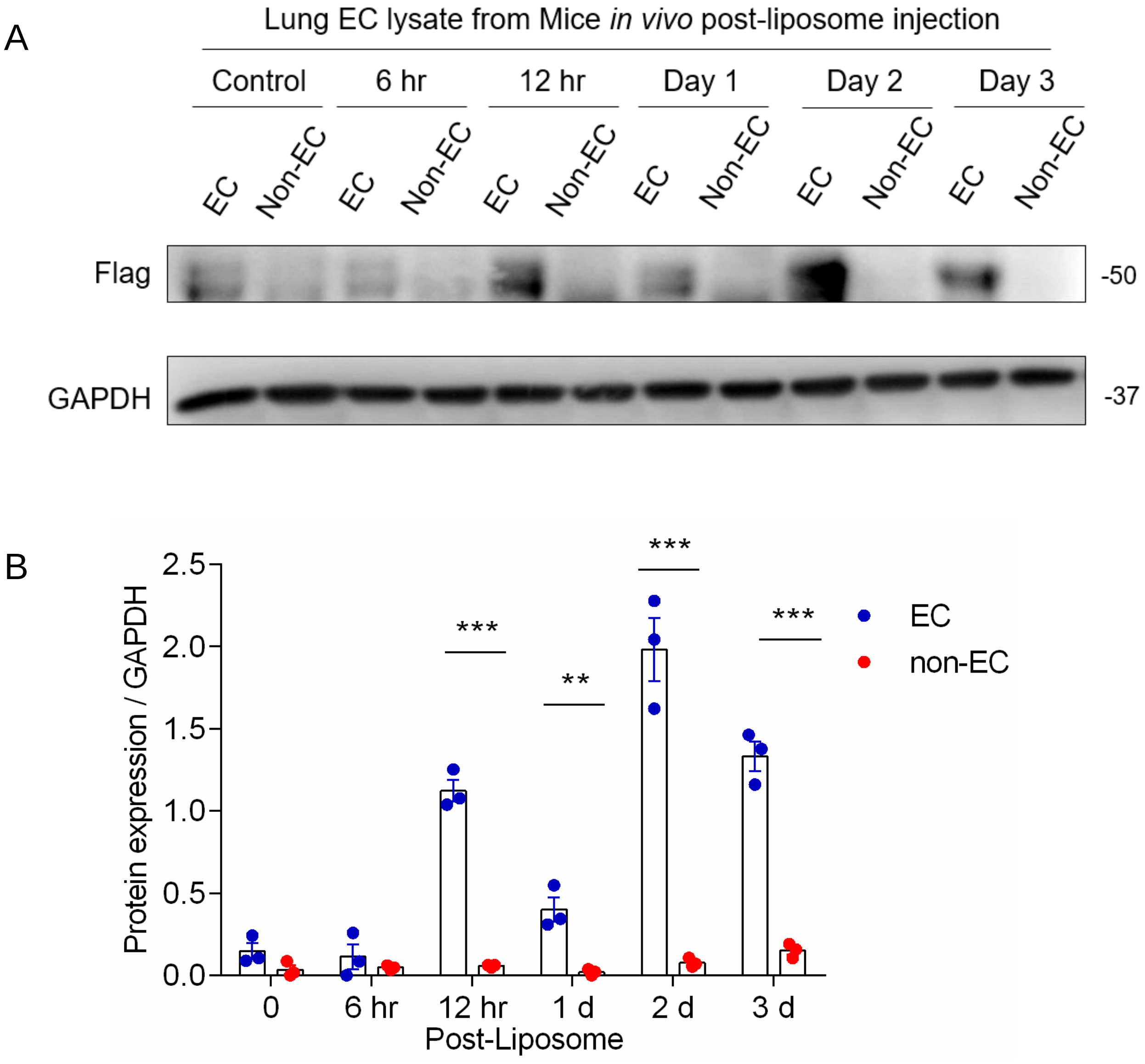

Supplementary Figure 7. Liposomal delivery of a plasmid with the endothelial-specific VE-cadherin promoter results in EC-specific expression within 2 days. (A) Immunoblotting for the Flag tag and loading control in isolated lung ECs and non-ECs from mice post-treatment of liposome-Sox17 plasmid complex at different time points (0, 6hr, 12hr, 1 day, 2 day and 3 day) and (B) its quantification. n = 3. **P < 0.01 and ***P < 0.001. Data are shown as mean ± SEM. Analysis is performed using two-way ANOVA with Bonferroni post-tests.

Supplementary Figure 8

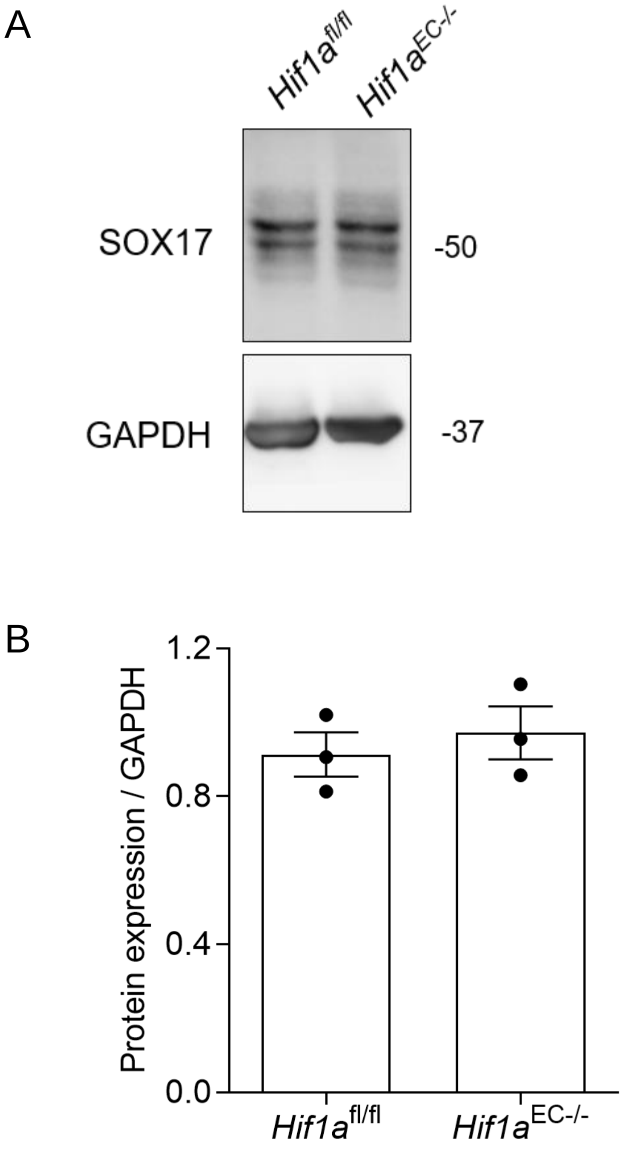

Supplementary Figure 8. EC-specific HIF-1α deletion in mice does not affect Sox17 expression. (A) Immunoblot of Sox17 protein in isolated lung ECs from HIF-1α fl/fl and HIF-1αEC-/- mice at baseline and (B) its quantification shows no significant difference in expression level. n=3. Data are shown as mean ± SEM. Analysis is performed using two-tailed Student’s t test.

Supplementary Figure 9

A

B

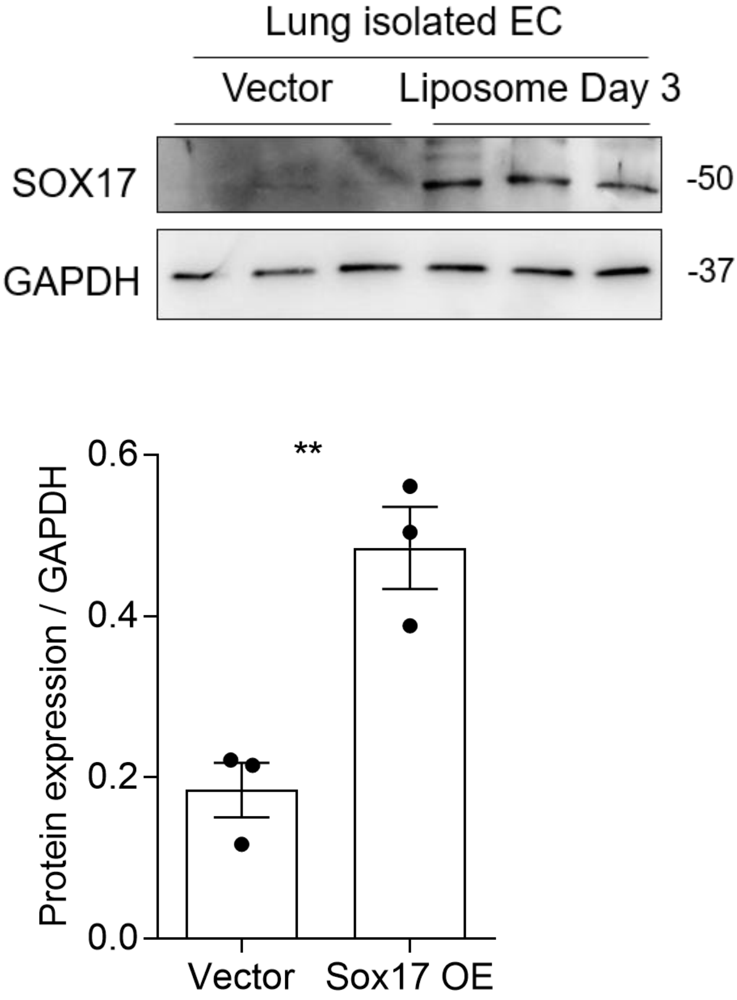

Supplementary Figure 9. Liposomal delivery increase Sox17 expression in lung ECs of mice. (A) Immunoblot of Sox17 protein in isolated lung ECs from mice treated with liposome-Vector and Sox17 plasmid complex and (B) its quantification. n=3. Data are shown as mean ± SEM. Analysis is performed using two-tailed Student’s t test.

Supplementary Figure 10

A

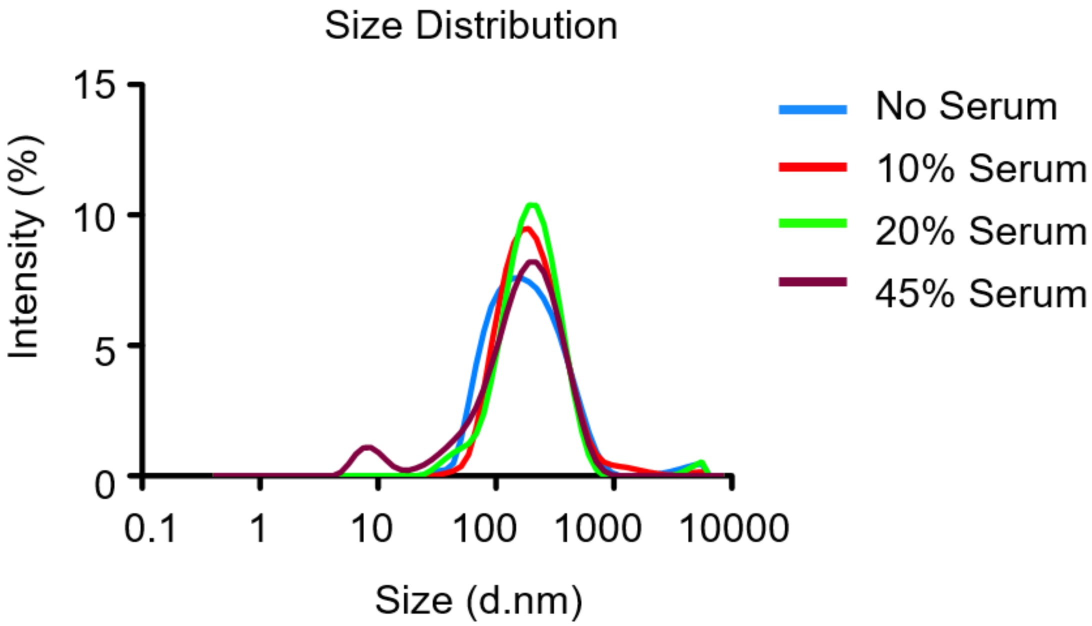

B

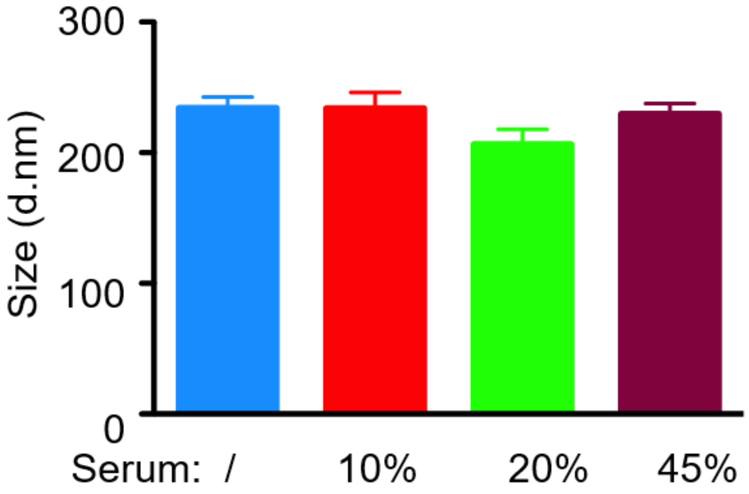

Supplementary Figure 10. Size characterization of liposomes indicates no large aggregate formation. (A and B) Serum was first ultracentrifuged for 2 hr at 140,000 g (Optima TLX Ultracentrifuge, Beckman Coulter) to remove extracellular vesicles that might interfere with size measurements. Liposomes were then prepared by combining 100 μl liposomes with 900 μl of varying serum fractions (no serum, 10%, 20% and 45% serum in 5% glucose buffer). After 5 min incubation, dynamic light scattering (Zetasizer Nano ZS, Malvern) was used to determine liposome size. Average sizes were similar for all groups and we did not see the formation of large aggregates. n = 3.

Supplementary Figure 11

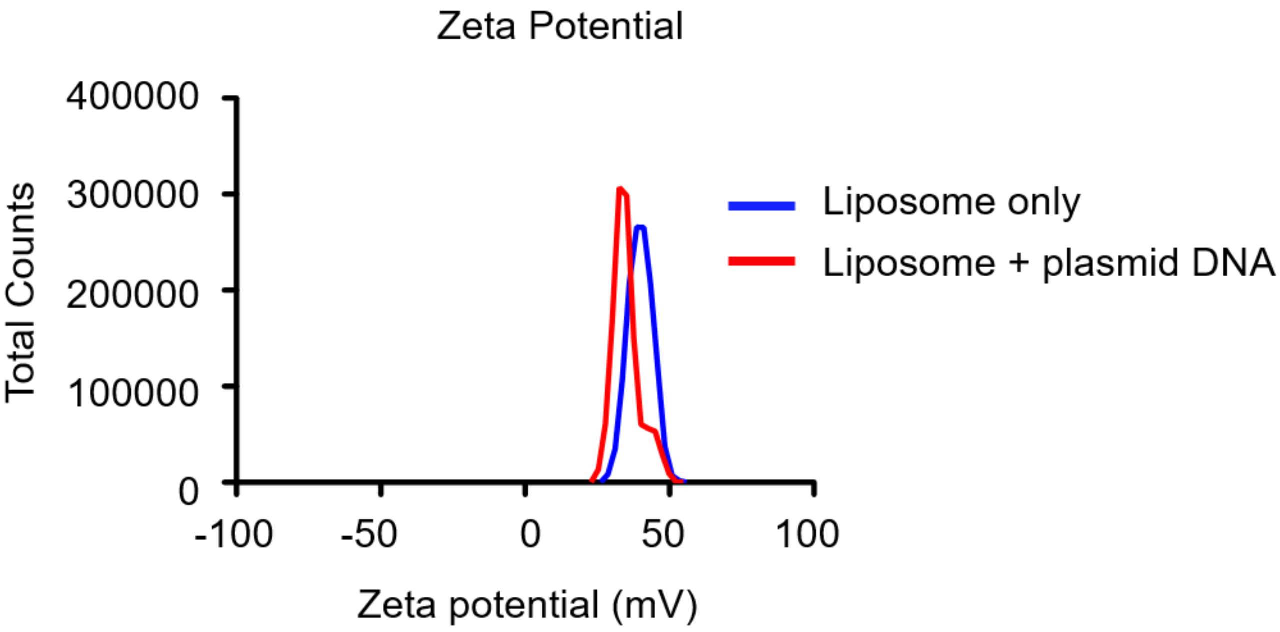

Supplementary Figure 11. Zeta potential characterization of liposomes indicates their cationic nature. Zeta potential of liposomes was 39.8 ± 0.2 mV and decreased to 34.8 ± 0.2 mV after adding plasmid DNA. These data confirm the cationic nature of liposomes even after adding DNA. n = 3.

Supplementary Figure 12

A

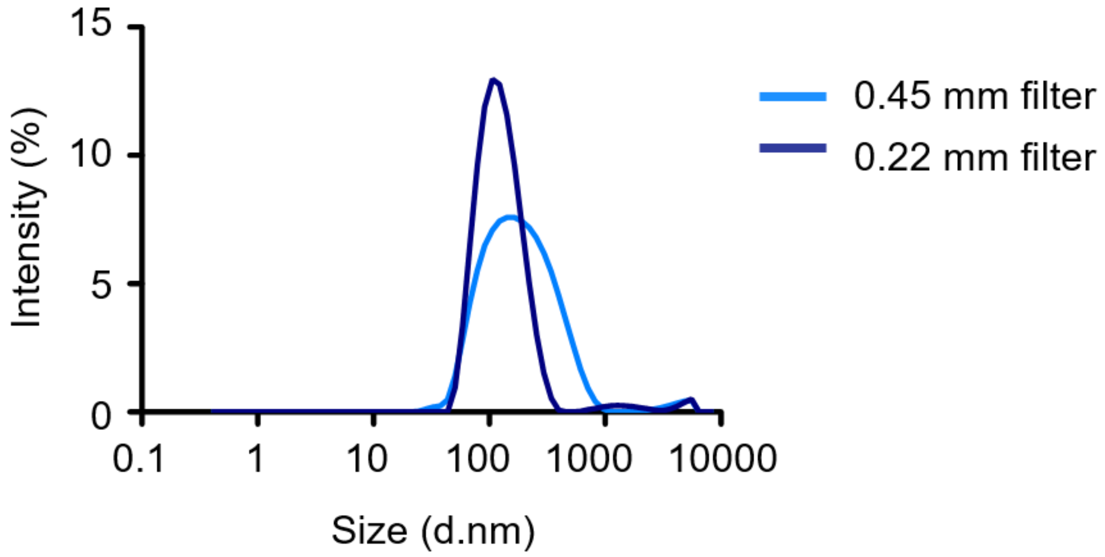

B

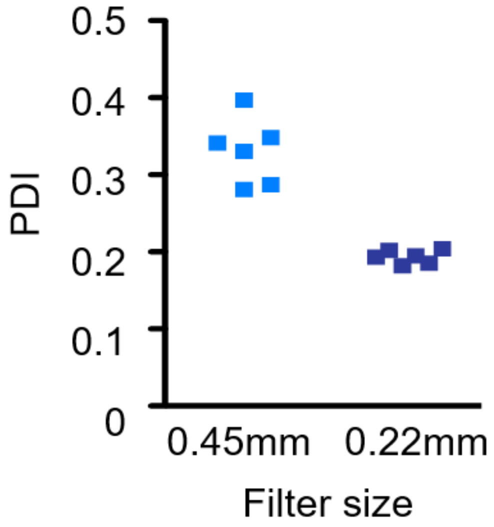

Supplementary Figure 12. Polydispersity index (PDI) characterization of liposomes. (A) Dynamic light scattering was used to determine the hydrodynamic diameter of liposomes prepared with a 0.45mm filter (which is used for all other experiments in this manuscript) and a 0.22mm filter. There is clear narrowing of the peak by using the 0.22mm filter. (B) Liposomes prepared using a 0.45mm filter have a PDI between 0.29 and 0.40.

Supplementary Figure 13

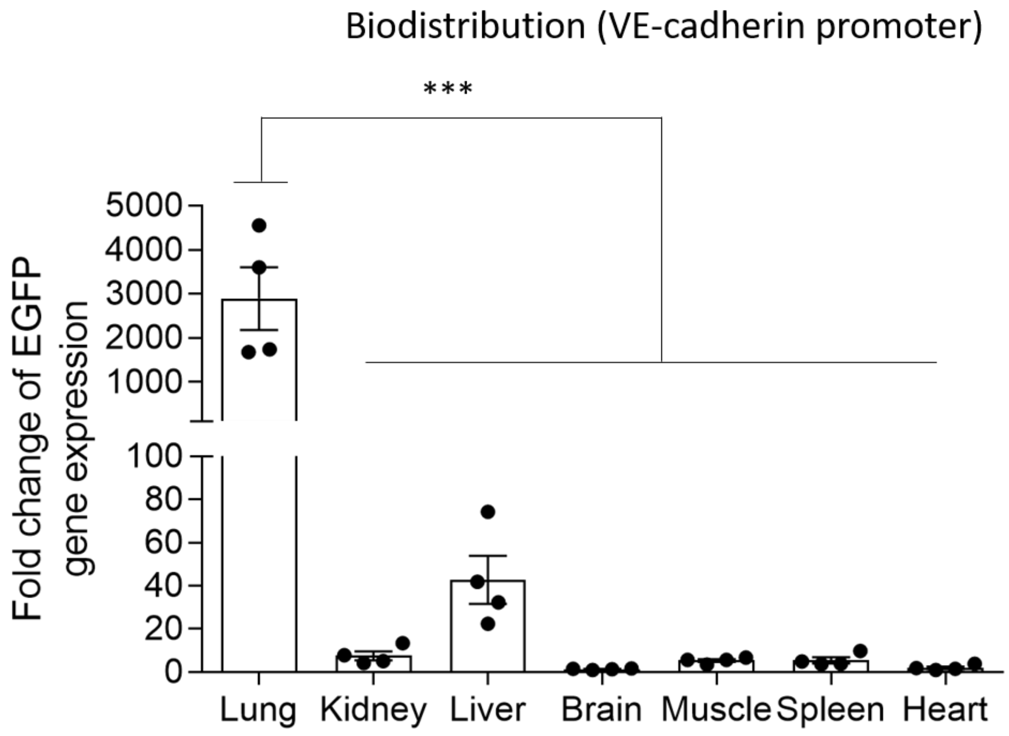

Supplementary Figure 13. Organ biodistribution of transgene expression delivered by injected liposome. Liposome loaded pCDNA-VE-cad promoter-EGFP plasmid is injected into the wildtype mice and then different organs were harvested 1 day later for RNA extraction. qPCR analysis of EGFP level (fold change compared to the baseline control) suggest that lung is the organ with highest transgene overexpression. n = 4. ***P < 0.001. Data are shown as mean ± SEM. Analysis is performed by one-way ANOVA with Dunnett post-test.

Supplementary Table 1. Mouse body weight for Evans blue transvascular albumin permeability experiments in Figure 3D, E & J and 6F. There is no significant change in body weight in mice treated with liposome + DNA or Vector, and pre-/post-LPS challenge.

Figure 3D	*Sox17*$^{fl/fl}$				*Sox17*$^{EC-/-\ (Endo\text{-}Scl\text{-}cre)}$			
PBS	23.4	17.5	20	25.6	19.8	21.8	19.5	22.2
LPS 1d	19.9	18.1	17.7	20.6	17.9	20.6	22.1	18.7
LPS 3d	19.6	18.6	17.2	24	14.1	18.7	13.6	21.8
LPS 5d	25.1	23.2	20.4	22.7	19.5	22.7	24	19

Figure 3E	*Sox17*$^{fl/fl}$				*Sox17*$^{EC-/-\ (CDH5\text{-}cre)}$			
PBS	18.3	24.3	25.6	19	24	25	23.2	20
LPS 1d	20.6	23.6	21.2	19.7	19	23	21.5	20.3
LPS 3d	21.6	18.6	19	20.6	17.6	20	22.9	19.7
LPS 5d	20.1	17.7	22.5	17.9	20.6	19.6	20.8	23.7

Figure 3J	Sox17$^{EC-/-}$ + Vector					Sox17$^{EC-/-}$ + Sox17 OE				
PBS	22.5	28	23.6	20	23.2					
LPS 3d	20.7	19.3	21	19.2	17.6	21.4	16.5	25	19.8	18.6

Figure 6F	WT + Vector					WT + Sox17 OE				
PBS	18	18.4	19	18.1	23.7	24	19.3	23.6	21.1	24
LPS 3d	19.1	22.5	17.6	22.1	22	20.7	22.1	22.2	19.4	19.7

Supplementary Table 2. List of primers used in this study.

Target gene		Primer sequence
PPIA	Fw	GGCAAATGCTGGACCAAACAC
	Rv	TTCCTGGACCCAAAACGCTC
Sox7	Fw	GGAAAGTCATGGAAGGCGCT
	Rv	GAGGCGCTTGCCTTGTTTC
Notch1	Fw	CTCCGTTACATGCAGCAGTT
	Rv	CCAGGATCAGTGGAGTTGTG
Kit	Fw	GGCCTCACGAGTTCTATTTACG
	Rv	GGGGAGAGATTTCCCATCACAC
Cdh5	Fw	GTCGATGCTAACACAGGGAATG
	Rv	AATACCTGGTGCGAAAACACA
Ccnd1	Fw	TGAGGAGCAGAAGTGCGAAGA
	Rv	CAAGGGAATGGTCTCCTTCATC
Klf2	Fw	GAGCCTATCTTGCCGTCCTTT
	Rv	CACGTTGTTTAGGTCCTCATCC
Sox18	Fw	TTTCCCAATCCTCTGTCACCACCA
	Rv	ACTGGTCAAATTCGGTGAGGTCCA
Pecam1	Fw	CCACCTCGAAAAGCAGGTCT
	Rv	CATCCCAGGGGGCTTGATTT
Atxn1	Fw	GTTACTCAGGAGGCAGCAGTTA
	Rv	GTACCCAGGATCTCCATACTTTC
Etv2	Fw	GCGGAATTTGGTTTCTATTTCCCT
	Rv	TTCCAGCAGCCTTCTACGTTC
Ccne1	Fw	AATTGGGGCAATAGAGAAGAGGT
	Rv	AGAAGTCCTGTGCCAAGTAGAA
Cd34	Fw	ATCCCCATCAGTTCCTACCAAT
	Rv	TGGTGTGGTCTTACTGCTGTC
Vegfr2	Fw	TTTGGCAAATACAACCCTTCAGA
	Rv	GCAGAAGATACTGTCACCACC
Sox17	Fw	ACCTACACTTACGCTCCAGTC
	Rv	GCCGTAGTACAGGTGCAGAG
Sox17 BS1	Fw	AGAAATGGGGGTCTCACTGTT
	Rv	GGTGGCTCACGCCTTTAATC
Sox17 BS2	Fw	ACATAGCAAGACCCTATCCCTAC
	Rv	CTCCTCTCCACAGCTCCTCT
Sox17 BS3	Fw	GGGAGTAGATAGTTGCCATCTG
	Rv	CTAAGTCCTTCAAAGTATCATCTCAA
Sox17 BS4	Fw	GCATTTACTGTGTGCCAGGT
	Rv	TGCCACAATTATCCTGTTTCAC
HIF-1α HRE1	Fw	GGGCAGGTGTAGCCTTG
	Rv	ACAGATGTGGCCAATGGAG
HIF-1α HRE2	Fw	GGTTGGACTGGGACGTG
	Rv	GGTCCCGCGTCGTTAG
HIF-1α HRE3	Fw	ACTGGGACGTGGGACT
	Rv	CCCGCGTCGTTAGGC
