# Supplementary material for: Sox17 is required for endothelial regeneration following inflammation-induced vascular injury
Source: Nat Commun. 2019 May 9;10:2126. doi: 10.1038/s41467-019-10134-y (PMC6509327; doi:10.1038/s41467-019-10134-y)
Supplement: Supplementary file 2 — Description of Additional Supplementary Files [file 41467_2019_10134_MOESM2_ESM.docx]

**Description of Supplementary Files**

**File Name:** Supplementary Movie 1 .

**Description:** 3D structure of mTmG-Scl-Cre mice lung in 2-photon microscopy at baseline. Red (tdTomato) indicates non-ECs and green (EGFP) indicates ECs. The Movie was constructed using Imaris software and recorded at 24 frames per second.

**File Name:** Supplementary Movie 2.

**Description:** 3D structure of mTmG-Scl-Cre mice lung under 2-photon microscopy at post-LPS day 1. Red (tdTomato) indicates non-ECs and green (EGFP) indicates ECs. The Movie was constructed using Imaris software and recorded at 24 frames per second.
