# Supplementary material for: Sox17 is required for endothelial regeneration following inflammation-induced vascular injury
Source: Nat Commun. 2019 May 9;10:2126. doi: 10.1038/s41467-019-10134-y (PMC6509327; doi:10.1038/s41467-019-10134-y)
Supplement: Supplementary file 5 — Reporting Summary [file 41467_2019_10134_MOESM5_ESM.pdf]

nature research

Corresponding author(s): Asrar B. Malik

Last updated by author(s): Mar 7, 2019

Reporting Summary

Nature Research wishes to improve the reproducibility of the work that we publish. This form provides structure for consistency and transparency in reporting. For further information on Nature Research policies, see Authors & Referees and the Editorial Policy Checklist.

Statistics

For all statistical analyses, confirm that the following items are present in the figure legend, table legend, main text, or Methods section.

n/a	Confirmed	
☐	☒	The exact sample size (*n*) for each experimental group/condition, given as a discrete number and unit of measurement
☒	☐	A statement on whether measurements were taken from distinct samples or whether the same sample was measured repeatedly
☐	☒	The statistical test(s) used AND whether they are one- or two-sided<br>*Only common tests should be described solely by name; describe more complex techniques in the Methods section.*
☒	☐	A description of all covariates tested
☐	☒	A description of any assumptions or corrections, such as tests of normality and adjustment for multiple comparisons
☐	☒	A full description of the statistical parameters including central tendency (e.g. means) or other basic estimates (e.g. regression coefficient) AND variation (e.g. standard deviation) or associated estimates of uncertainty (e.g. confidence intervals)
☐	☒	For null hypothesis testing, the test statistic (e.g. *F*, *t*, *r*) with confidence intervals, effect sizes, degrees of freedom and *P* value noted<br>*Give P values as exact values whenever suitable.*
☒	☐	For Bayesian analysis, information on the choice of priors and Markov chain Monte Carlo settings
☒	☐	For hierarchical and complex designs, identification of the appropriate level for tests and full reporting of outcomes
☒	☐	Estimates of effect sizes (e.g. Cohen's *d*, Pearson's *r*), indicating how they were calculated

Our web collection on statistics for biologists contains articles on many of the points above.

Software and code

Policy information about availability of computer code

Data collection	No software was used.
Data analysis	No software was used.

For manuscripts utilizing custom algorithms or software that are central to the research but not yet described in published literature, software must be made available to editors/reviewers. We strongly encourage code deposition in a community repository (e.g. GitHub). See the Nature Research guidelines for submitting code & software for further information.

Data

Policy information about availability of data

All manuscripts must include a data availability statement. This statement should provide the following information, where applicable:

- Accession codes, unique identifiers, or web links for publicly available datasets
- A list of figures that have associated raw data
- A description of any restrictions on data availability

The authors declare that all relevant data supporting the findings of this study are available within the paper and its supplementary information files or from the authors upon reasonable request. Please contact Asrar B. Malik (abmalik@uic.edu) for any inquiries.

Field-specific reporting

Please select the one below that is the best fit for your research. If you are not sure, read the appropriate sections before making your selection.

☒ Life sciences ☐ Behavioural & social sciences ☐ Ecological, evolutionary & environmental sciences

For a reference copy of the document with all sections, see nature.com/documents/nr-reporting-summary-flat.pdf

Life sciences study design

All studies must disclose on these points even when the disclosure is negative.

Sample size	Based on our experience, we expect changes in the gene/protein expression and function measurements to be detected with 3 mice per group, so the effect size is was determined as n = 3 or n > 3.
Data exclusions	No samples or animals were excluded from the analysis.
Replication	All attempts at replication were successful.
Randomization	Mice were randomly assigned to treatment arms with approximately equivalent numbers in each group.
Blinding	No blinding was done for animal experiments, since the functional readout is determined by standard assay and not subjective scoring.

Reporting for specific materials, systems and methods

We require information from authors about some types of materials, experimental systems and methods used in many studies. Here, indicate whether each material, system or method listed is relevant to your study. If you are not sure if a list item applies to your research, read the appropriate section before selecting a response.

Materials & experimental systems

n/a	Involved in the study	
☐	☒	Antibodies
☐	☒	Eukaryotic cell lines
☒	☐	Palaeontology
☐	☒	Animals and other organisms
☒	☐	Human research participants
☒	☐	Clinical data

Methods

n/a	Involved in the study	
☒	☐	ChIP-seq
☐	☒	Flow cytometry
☒	☐	MRI-based neuroimaging

Antibodies

Antibodies used	The company name and catalog number of all antibodies are provided in the method section after each antibody when mentioned.
Validation	Antibody validation and relevant citations are listed on manufacturer's website for each antibody.

Eukaryotic cell lines

Policy information about cell lines

Cell line source(s)	293T cells (ATCC #CRL-3216; Clontech # 632180)
Authentication	The cell line was not authenticated.
Mycoplasma contamination	The cell line was not tested for mycoplasma contamination.
Commonly misidentified lines (See ICLAC register)	No commonly misidentified lines used.

Animals and other organisms

Policy information about studies involving animals; ARRIVE guidelines recommended for reporting animal research

Laboratory animals	C57BL/6 mice (Strain # 027) were purchased from Charles River Laboratory. ROSAmT/mG mice (Jackson Lab, Stock # 007576) were crossed with endothelial-enhanced SCLScl-CreERT2 mice 31 (provided by Dr. Joachim Göthert). Mice were gauge fed with Tamoxifen (Sigma #T5648; 20 mg/ml in corn oil) for consecutive five days and then rest for four weeks to induce EC-specific membrane EGFP expression. Afterward, mice were challenged with sub-lethal LPS (Sigma #L2630) i.p. 12 mg/kg and then sacrificed at different time points for tissue harvest. Sox17fl/fl mice (Jackson Lab, Stock # 027712) were crossed with endothelial-enhanced SCLScl-CreERT2 mice and CDH5Cdh5-CreERT2 (provided by Dr. Ralf Adams) mice 36, respectively. Mice were gauge fed with Tamoxifen (20 mg/ml in corn oil) for consecutive five days and then rest for four weeks to induce EC-specific Sox17 protein deletion. Afterward, Sox17EC-/- and control (Sox17fl/fl Cre negative mice with tamoxifen) mice were challenged with sub-lethal LPS 8 mg/kg i.p. and then sacrificed at different time points for tissue harvest. HIF-1αHif1afl/fl mice (Jackson Lab, Stock

007561) were crossed with Tie2-CreERT2 mice 78 (Jackson Lab, Stock #2450312). Mice were gauge fed with Tamoxifen (20 mg/ml in corn oil) for consecutive five days and then rest for four weeks to induce EC-specific HIF-1α protein deletion. Afterward, HIF-1αHif1aEC-/- and control (HIF-1αHif1afl/fl Cre negative mice with tamoxifen) mice were challenged with sub-lethal LPS 12 mg/kg i.p. and then sacrificed at different time points for tissue harvest. All experiments were performed with 8–12-wk-old animals and were performed by using age- and sex-matched groups. All animals were on a C57BL/6 background. The mice were maintained in a pathogen-free environment in University of Illinois at Chicago Animal Care Facility (Biological Resource Center).

Wild animals	The study did not involve wild animals.
Field-collected samples	The study did not involve samples collected from the field.
Ethics oversight	All animal experiments were conducted in accordance with NIH guidelines for the Care and Use of Laboratory Animals and were approved by the IACUC of the University of Illinois.

Note that full information on the approval of the study protocol must also be provided in the manuscript.

Flow Cytometry

Plots

Confirm that:

- [x] The axis labels state the marker and fluorochrome used (e.g. CD4-FITC).
- [x] The axis scales are clearly visible. Include numbers along axes only for bottom left plot of group (a 'group' is an analysis of identical markers).
- [ ] All plots are contour plots with outliers or pseudocolor plots.
- [x] A numerical value for number of cells or percentage (with statistics) is provided.

Methodology

Sample preparation	Flushed mice lung was minced and digested with 5 ml Type 1 collagenase I (2 mg/ml in PBS) at 37ºC water bath for 1 hour. Mixtures were titrated with #18 needles and then pipetted through a 40 μm disposable cell strainer. After centrifuge 300 g for 5 minutes and wash with PBS, the Isolated cells were treated with red blood cell lysis buffer (eBioscience) for 5 minutes on ice to lyse red blood cells. Afterwards, remaining cells were incubated with anti-mouse CD16/CD32 (1:50, BD Pharmingen #553142) to block endogenous Fc for 10 minutes on ice. Then, cells were stained with antibodies including CD45-EF450 (1:2000, eBioscience #48-0451-82) and CD31-APC (1:100, eBioscience #17-0311-82) for 45 minutes at 4°C. After wash, the cells were resuspended in 500 μl buffer and analyzed on an LSRFortessa (BD Pharmingen) cell analyzer. Obtained data were analyzed by Summit software (Beckman Coulter).
Instrument	LSRFortessa (BD Pharmingen) cell analyzer
Software	Summit software (Beckman Coulter)
Cell population abundance	No cell sorting was conducted.
Gating strategy	The gating strategy is included in supplementary figure 1. FSC was determined to exclude platelets. SSC gate was determined to exclude cell aggregation. A negative Cre mice with antibody isotype staining was used as negative control for "positive" vs "negative" boundaries set up.

- [x] Tick this box to confirm that a figure exemplifying the gating strategy is provided in the Supplementary Information.
