# Supplementary material for: Sox17 is required for endothelial regeneration following inflammation-induced vascular injury
Source: Nat Commun. 2019 May 9;10:2126. doi: 10.1038/s41467-019-10134-y (PMC6509327; doi:10.1038/s41467-019-10134-y)

Full unedited gel for Figure 2B

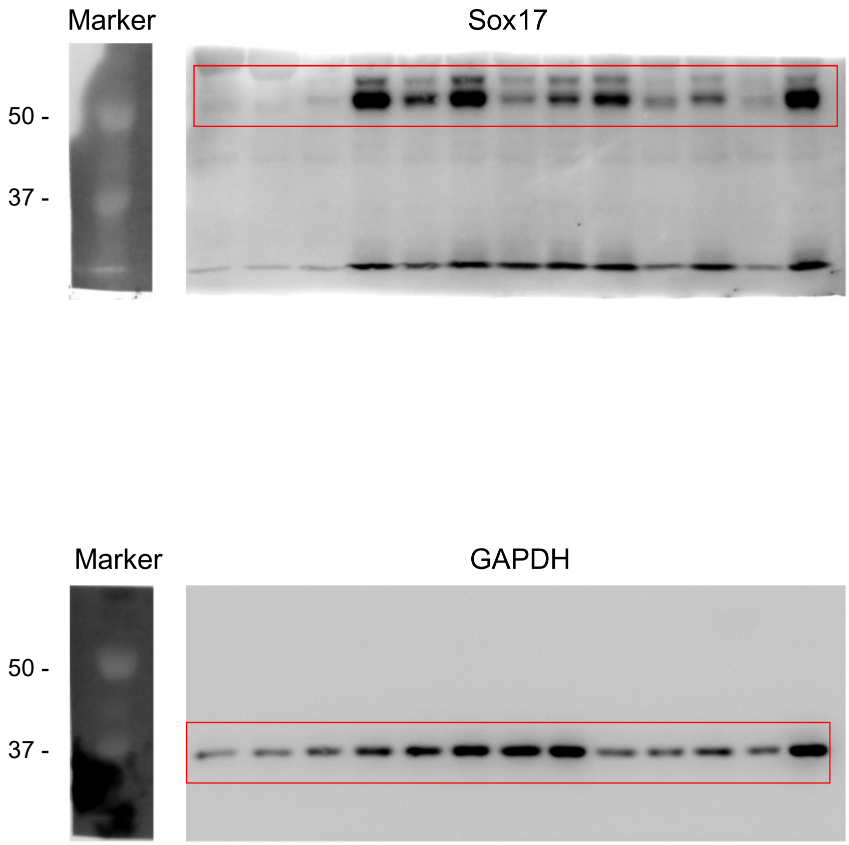

Full unedited gel for Figure 2D

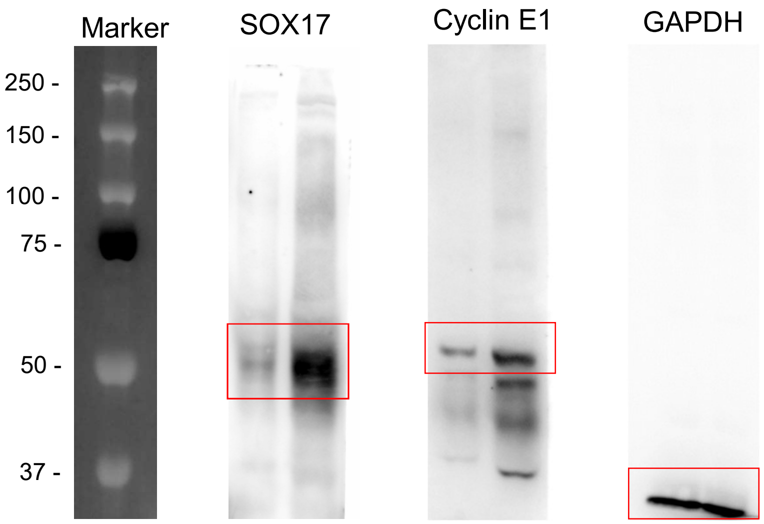

Full unedited gel for Figure 3B

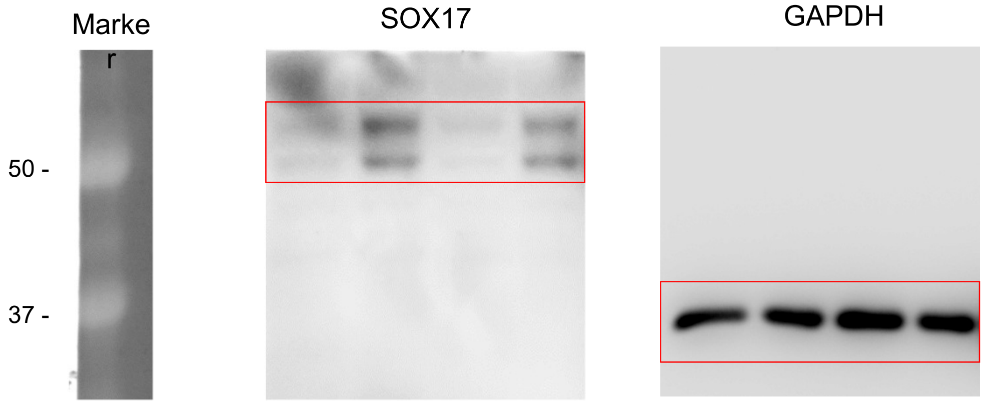

Full unedited gel for Figure 4B

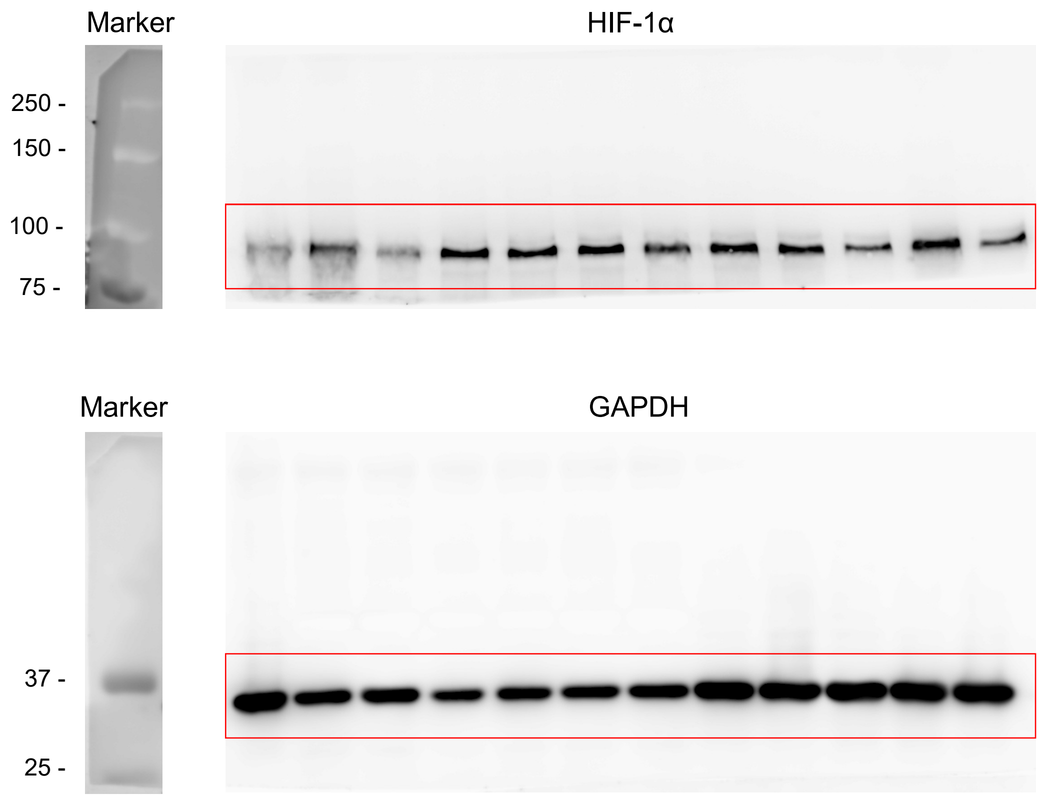

Full unedited gel for Figure 4D

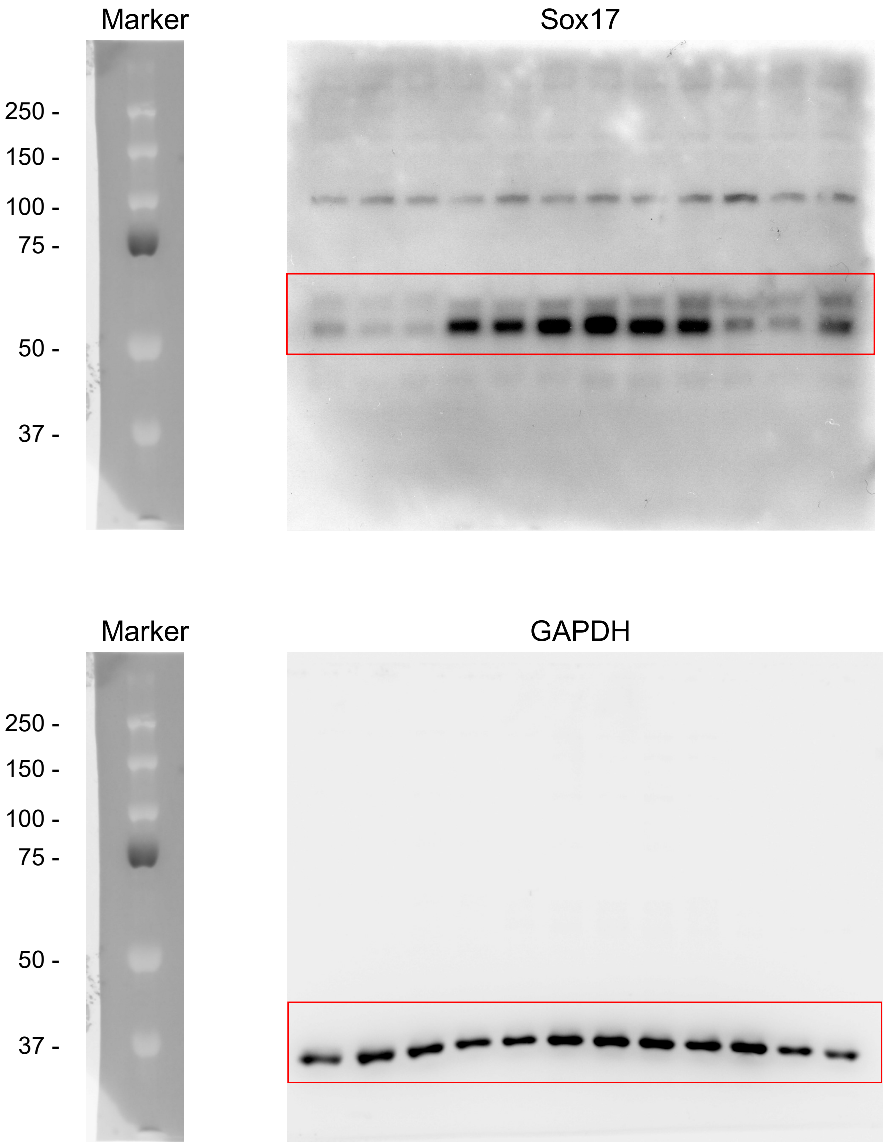

Full unedited gel for Figure 4F

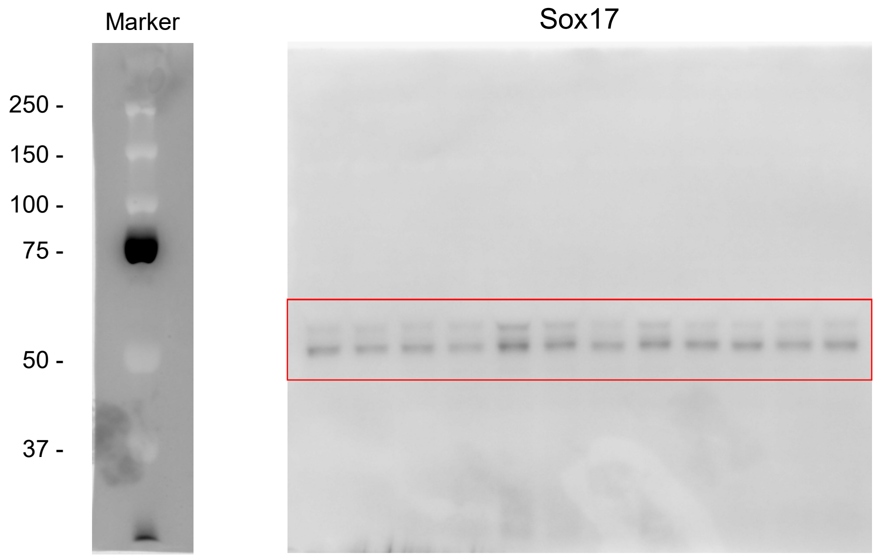


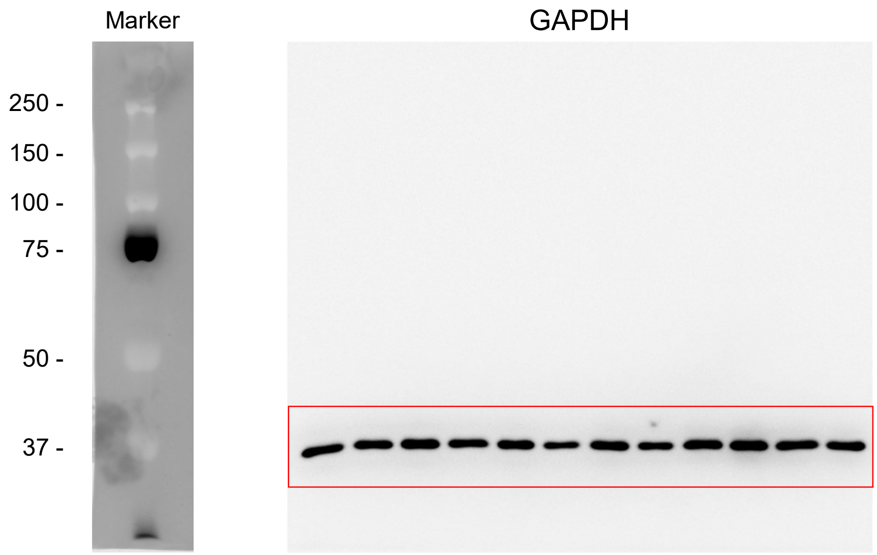

Full unedited gel for Figure 5A

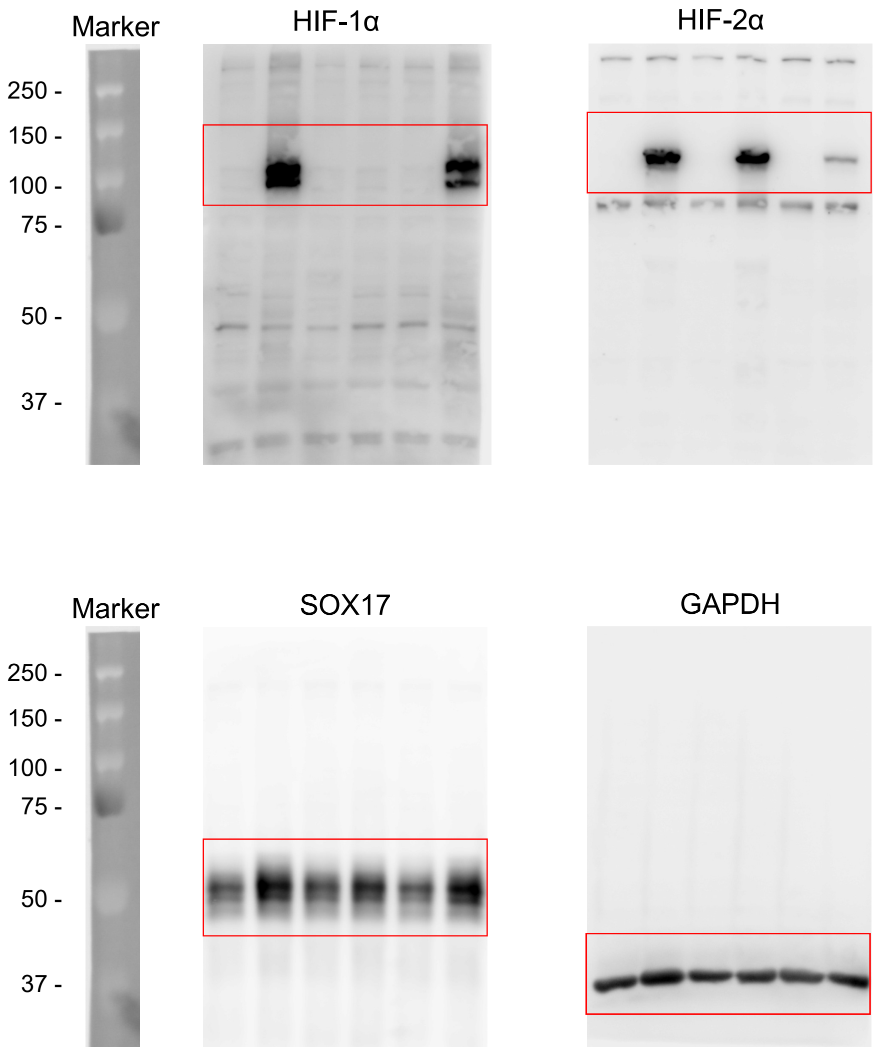

Full unedited gel for Figure 6C

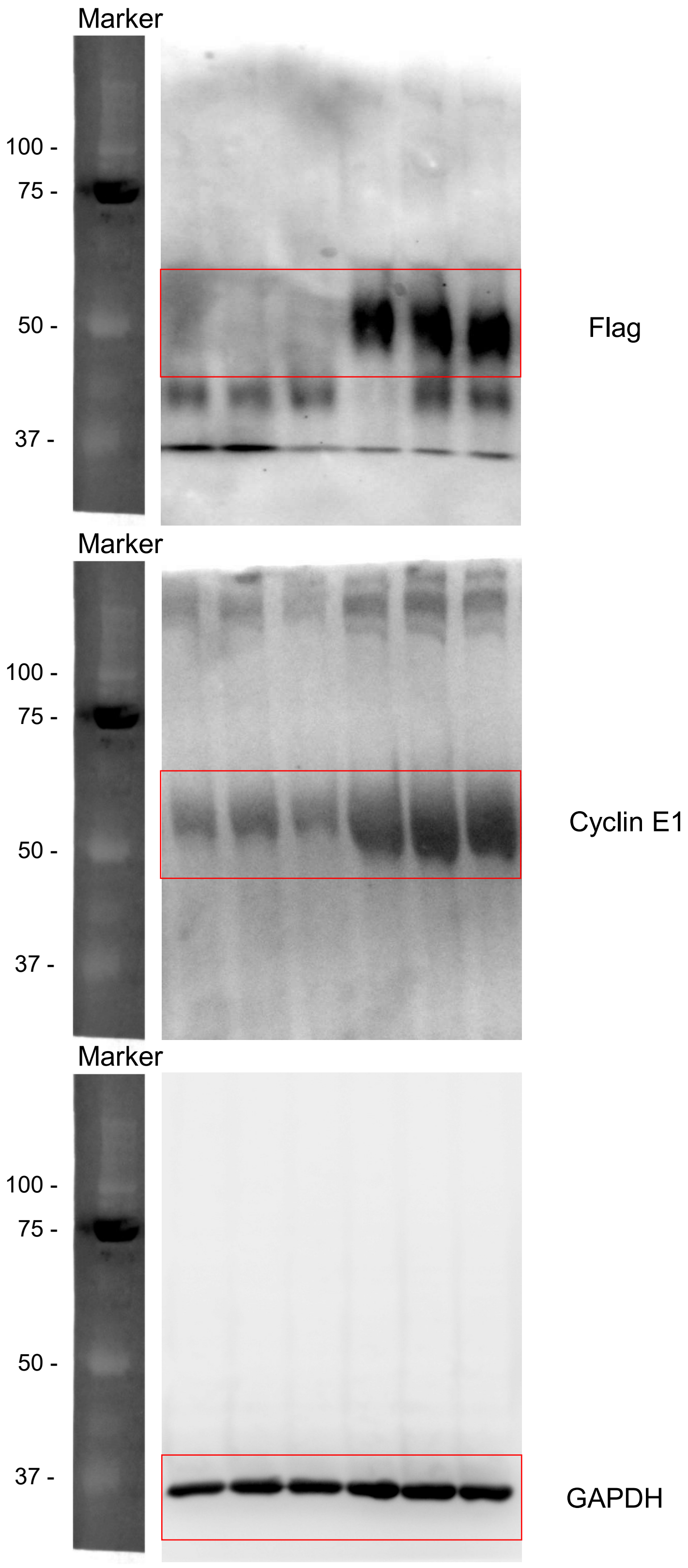

Full unedited gel for Supplementary Figure 2A

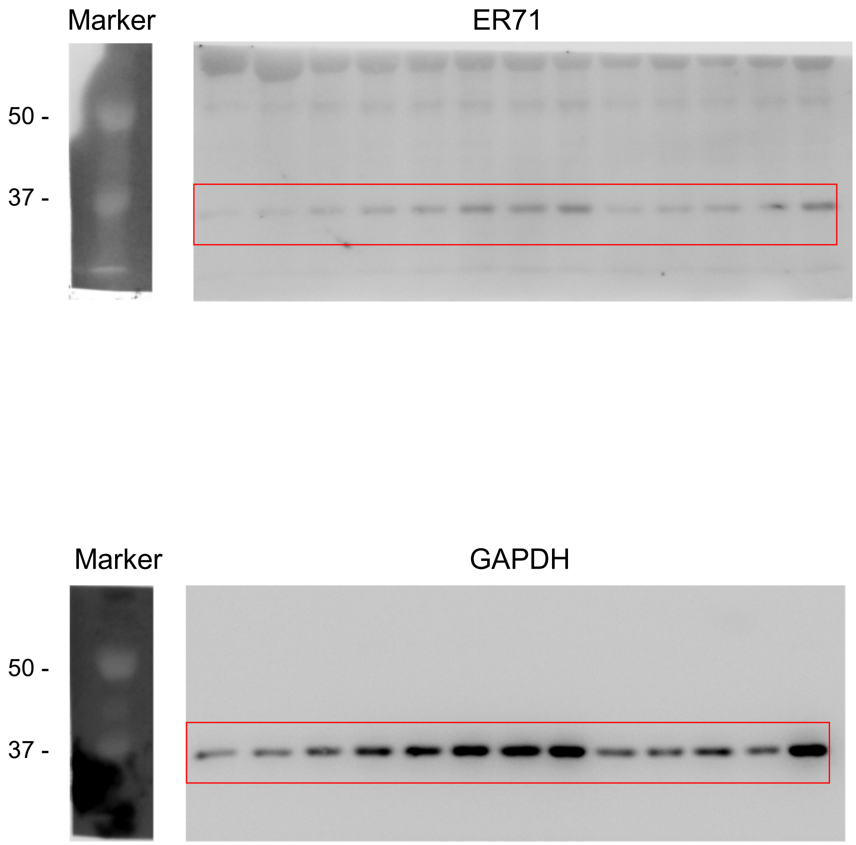

Full unedited gel for Supplementary Figure 7A

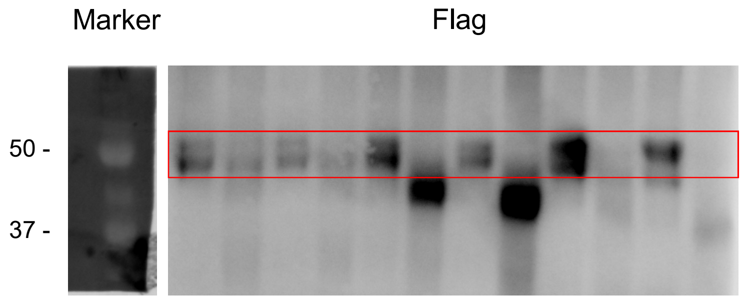


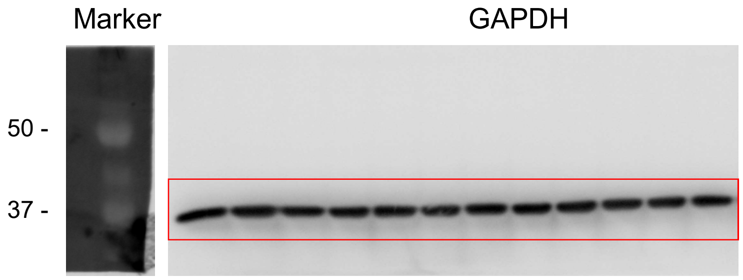

Full unedited gel for Supplementary Figure 8A

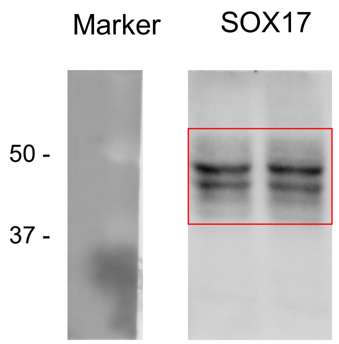


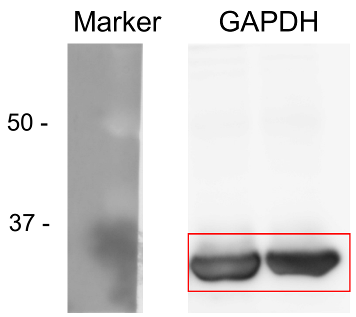

Full unedited gel for Supplementary Figure 9A

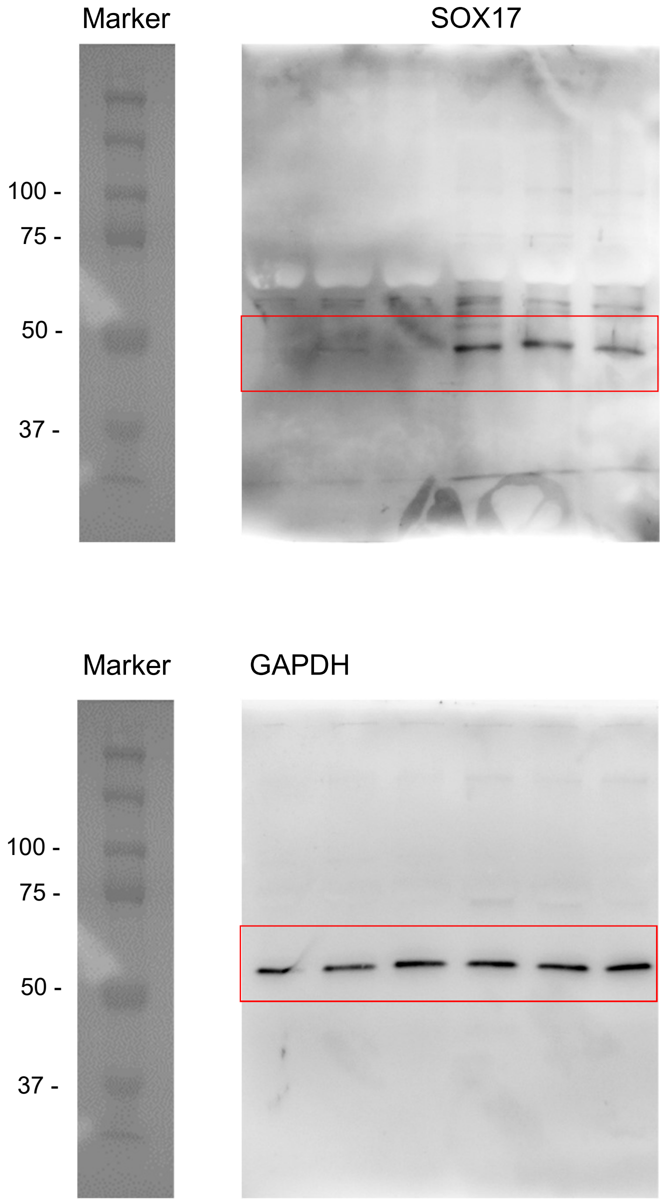

Supplement: Supplementary file 6 — Source Data [file 41467_2019_10134_MOESM6_ESM.pdf]
